# Interaction with mammalian enteric viruses alters outer membrane vesicle production and content by commensal bacteria

**DOI:** 10.1002/jev2.12172

**Published:** 2022-01-04

**Authors:** Chanel A. Mosby, Sutonuka Bhar, Matthew B. Phillips, Mariola J. Edelmann, Melissa K. Jones

**Affiliations:** ^1^ Microbiology and Cell Science Department IFAS University of Florida Gainesville Florida USA; ^2^ Department of Molecular Genetics and Microbiology College of Medicine University of Florida Gainesville Florida USA

**Keywords:** bacterial extracellular vesicles, *Bacteroides thetaiotaomicron*, commensal bacteria, commensal bacteria transcriptomics, *Enterobacter cloacae*, human norovirus, *Lactobacillus acidophilus*, microbiome, murine norovirus, outer membrane vesicles, proteomics, RNA‐seq

## Abstract

Intestinal commensal bacteria contribute to maintaining gut homeostasis. Disruptions to the commensal flora are linked to the development and persistence of disease. The importance of these organisms is further demonstrated by the widespread ability of enteric viruses to exploit commensal bacteria to enhance viral infection. These viruses interact directly with commensal bacteria, and while the impact of this interaction on viral infection is well described for several viruses, the impact on the commensal bacteria has yet to be explored. In this article, we demonstrate, for the first time, that enteric viruses alter the gene expression and phenotype of individual commensal bacteria. Human and murine norovirus interaction with bacteria resulted in genome‐wide differential gene expression and marked changes in the surface architecture of the bacterial cells. Furthermore, the interaction of the virus with bacteria led to increased production of smaller outer membrane vesicles (OMVs). Enhanced production of smaller vesicles was also observed when noroviruses were incubated with other commensal bacteria, indicating a potentially broad impact of norovirus interaction. The vesicle production observed in the in vivo model followed a similar trend where an increased quantity of smaller bacterial vesicles was observed in stool collected from virus‐infected mice compared to mock‐infected mice. Furthermore, changes in vesicle size were linked to changes in protein content and abundance, indicating that viral binding induced a shift in the mechanism of the OMV biogenesis. Collectively, these data demonstrate that enteric viruses induce specific changes in bacterial gene expression, leading to changes in bacterial extracellular vesicle production that can potentially impact host responses to infection.

## 1 INTRODUCTION

The intestinal microbiome has a profound impact on host health and disease. The commensal bacteria that comprise the microbiome are associated with various biological processes, including the acquisition of nutrients, regulation of intestinal permeability and modulation of host immune responses (Harris et al., 2017; Hooper & Gordon, 2001; Hooper et al., 2001; Kendall & Sperandio, 2016). These bacteria play an integral role in maintaining gut homeostasis, and their disruption is linked to the development and severity of chronic illness, including irritable bowel disease, Crohn's disease and ulcerative colitis (Ahmed et al., 2016; Wright et al., 2015). One of the regulatory mechanisms by which commensal bacteria modulate host responses and disease is the production of bacterial extracellular vesicles (bEVs)(7‐9). These nanosized, proteolipid vesicles are produced by both Gram‐negative and positive bacteria and are referred to, in this study, as outer membrane vesicles (OMVs) and cytoplasmic membrane vesicles (CMVs), respectively. OMVs are comprised of membrane‐integrated proteins, receptors and signalling molecules that can interact directly with other bacteria and host cells (Bhar et al., 2021). Internally, these vesicles contain cytoplasmic and periplasmic material that aid in transcytosis across epithelial barriers or modulation of host responses (Choi et al., 2017; Furuse et al., 2014), and changes in vesicle content are linked to shifts in vesicle size, which can alter their interaction with the host. OMVs may also contain genetic material depending on the mechanism of vesicle biogenesis (Choi et al., 2017; Nagakubo et al., 2020).

In addition to impacting host health and disease, the intestinal microbiome also alters infections caused by enteric viral pathogens. Specifically, the commensal gut flora enhances infection of several enteric viruses, including poliovirus, coxsackievirus, rotavirus and noroviruses (Baldridge et al., 2015; Jones et al., 2014; Kuss et al., 2011; Robinson et al., 2019; Uchiyama et al., 2014). All enteric viruses must interact with and pass through the gut flora to access host cells for infection, and many of these viruses are known to bind directly to commensal bacteria (Almand et al., 2017; Berger et al., 2017; Madrigal et al., 2020). To date, research investigating enteric virus interactions with commensal bacteria has focussed on how this interaction impacts viral infection (Berger et al., 2017; Erickson et al., 2018; Grau et al., 2020). While it is well established that enteric viral infection can alter the overall composition and diversity of the intestinal flora (Chen et al., 2020; Engevik et al., 2020; Jang et al., 2019; Singh et al., 2015), it is not yet known how the direct attachment of these viruses impacts specific bacterial species. Commensal bacteria respond rapidly and directly to external stimuli (Kendall & Sperandio, 2016; Singh et al., 2017). Specifically, exposure to external stimuli alters bacterial gene expression and activity through a broad network of signal transduction cascades (Fenchel, 2002; López‐Maury et al., 2008; Parkinson, 1993; Segall et al., 1982; Seshasayee et al., 2006). Consequently, we hypothesised that viral interaction with commensal bacterium alters bacterial gene expression, leading to changes in bacterial physiology.

To investigate this hypothesis, we tested the ability of two related enteric viruses, human (HNoV) and murine norovirus (MNV), to alter gene expression of a commensal bacterium, *Enterobacter cloacae*, which is bound by noroviruses and associated with the enhancement of human norovirus infection (Jones et al., 2015, 2014; Madrigal et al., 2020). Transcriptome analysis revealed that both viruses induced significant but divergent changes in gene expression. Among the differentially regulated genes found in common between the two viruses was a repertoire of genes associated with membrane stability and/or OMV formation. The use of scanning electron microscopy revealed changes in the surface architecture of *E. cloacae* in the presence of norovirus and increased surface appendages. Further investigation also revealed that both viruses increased the production of smaller vesicles by *E. cloacae* and two additional commensal bacteria. Bacterial vesicle production was also increased in mice when infected with MNV. Based on the link between vesicle size and content (Turner et al., 2018), we performed proteomic analysis on vesicles produced in the presence and absence of the virus. We found that virally‐induced vesicles possessed a decreased abundance of a specific protein subset indicative of a shift in the mechanism of OMV biogenesis. Together, these data reveal that enteric viruses can alter the physiological processes within the bacteria to which they are bound. Given the critical role OMVs play in host immune modulation and communication (Cañas et al., 2018; Maerz et al., 2018; Shen et al., 2012), the virus‐induced changes presented here may provide a novel mechanism by which the microbiome communicates with the host during enteric viral infection and may also provide a mechanism for enhancement of that infection.

## 2 RESULTS

### 2.1 Changes in *E. cloacae* gene expression upon interaction with noroviruses

**FIGURE 1:**
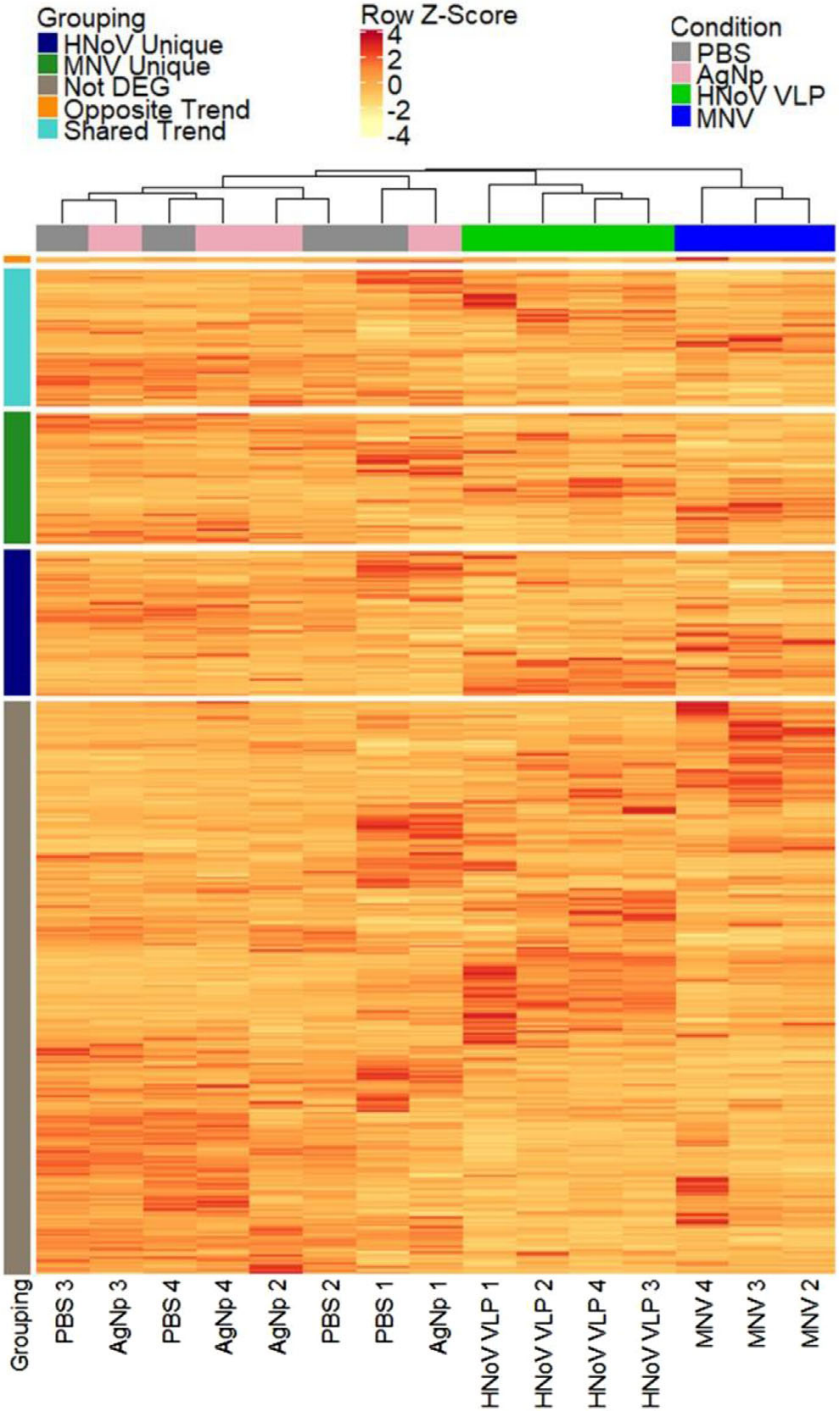
Altered *E. cloacae* gene expression correlates with viral exposure. Cultures of *E. cloacae* (10^8^ CFU/ml) were incubated with MNV‐1.CW3 (MOI = 0.1), HNoV GII.4 VLPs (0.1 mg/ml), AgNP (equivalent amount to HNoV) or PBS for 1 h at 37°C. Heatmap shows the clustering of all genes within sample types and distinct expression patterns between MNV, HNoV GII.4 and control cultures. Row slices correspond to differentially expressed genes that are unique to the HNoV condition (labeled “HNoV Unique”), unique to the MNV condition (labelled “MNV Unique”), not found to be differentially expressed (labelled “Not DEG”), inversely differentially expressed between MNV and HNoV conditions (labelled “Opposite Trend”), or up or down expressed in both MNV and HNoV (labelled “Shared Trend”). Rows were scaled with z‐scores for improved coloration and are based on the normalized counts

Whole transcriptome sequencing was performed to investigate the effects of viral exposure on *E. cloacae* gene expression. Bacterial attachment assays were performed (Madrigal et al., 2020) where *E. cloacae* were incubated with either phosphate‐buffered saline (PBS), MNV, HNoV GII.4 virus‐like particles (VLPs) or 40‐nm silver nanoparticles (AgNP). Nanoparticles were included to determine if changes in gene expression were due to viral interaction specifically or the result of bacterial interaction with nano‐size particles. Both norovirus strains induced a genome‐wide response by *E. cloacae* (Figure 1). The principal component analysis indicated distinct clustering of PBS and AgNp control samples and separation of MNV and HNoV samples from each other and the controls (Figure S1). One replicate of the MNV‐treated bacteria was discarded due to low mapping scores.

**FIGURE 2:**
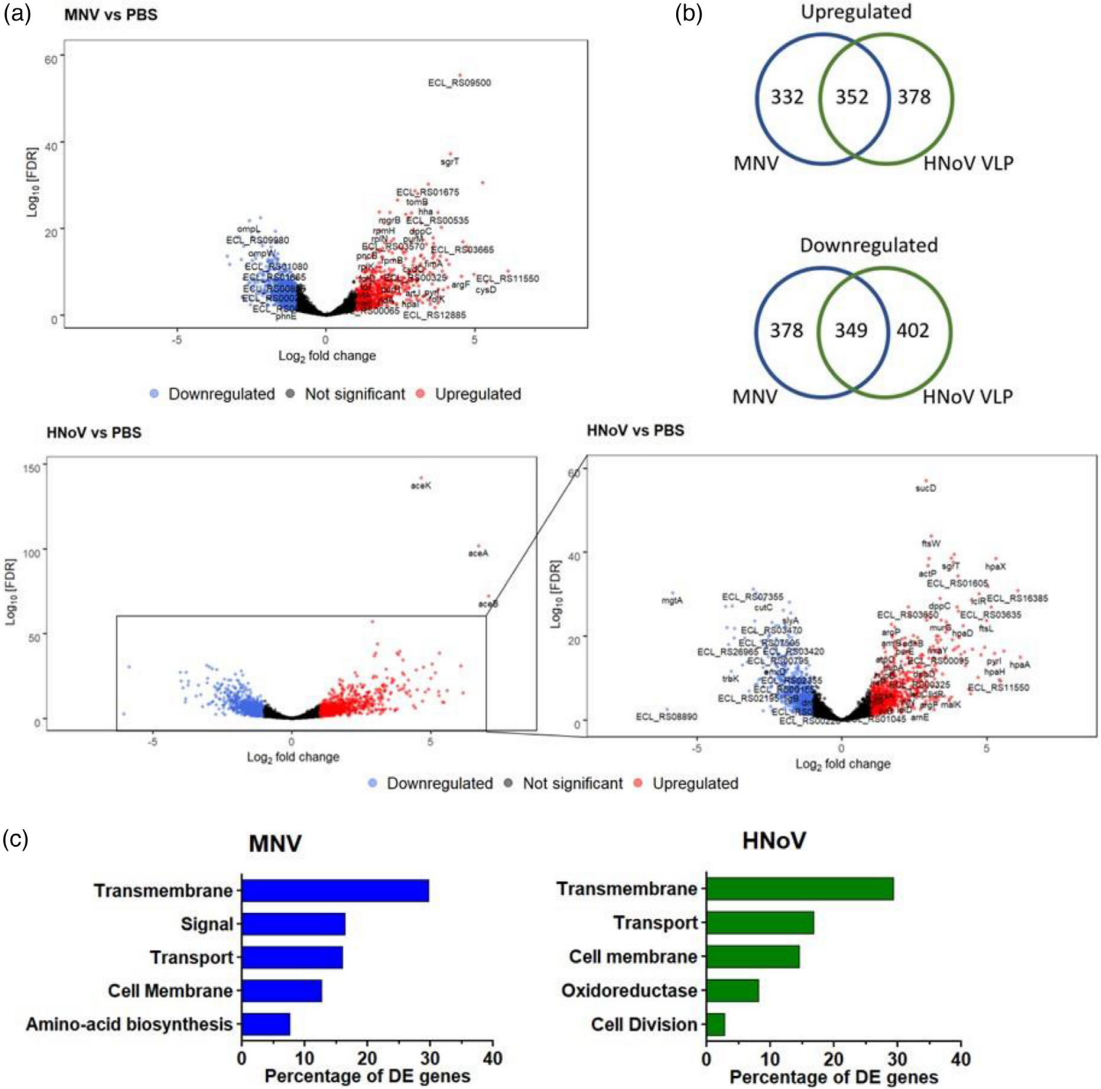
Incubation with murine norovirus and human norovirus VLPs alters gene expression of *E. cloacae*. Cultures of *E. cloacae* (10^8^ CFU/ml) were incubated with MNV‐1.CW3 (MOI = 0.1), HNoV GII.4 VLPs (0.1 mg/ml), AgNP (equivalent amount to HNoV) or PBS for 1 h at 37°C. a) Volcano plot showing the distinct transcriptional profiles for HNoV versus PBS and MNV versus PBS RNA‐sequencing results. Insert is a zoom of the HNoV plot excluding the extremely significantly expressed genes *aceA, aceB* and *aceK*. Fold‐change values and FDR‐adjusted *p*‐values were calculated using the DESeq2 R package and are from *n *= 3 (MNV) and *n* = 4 (PBS, HNoV, AgNp) biologically independent replicates. The FDR‐adjusted *p*‐value cutoff was set to < 0.05 and the log_2_ fold change cutoff was set to ±1. The volcano plot for AgNp versus PBS is not shown as only three genes were considered to be differentially regulated. b) The Venn diagrams show the similarities and differences of the differentially expressed genes (Log_2_ fold change of ±1 and FDR P‐value cut off < 0.05) when HNoV or MNV are attached to *E. cloacae*. c) Functional annotation of the significantly differentially express genes (Log 2‐fold change ±2) in *E. cloacae*. The differentially expressed genes were enriched in functional categories based on UniProtKB keyword annotations. The top five functional groups are ordered by the percentage of differential genes in the category, removing child categories, and those that did not meet the significance test (FDR < 0.05). Complete functional analysis results are in Dataset S1

Using an adjusted P‐value cut off of < 0.05 and a log_2_ fold change of ±1, there was a total of 1411 differentially expressed genes (DEG) in *E. cloacae* when incubated with MNV and 1481 DEGs when incubated with HNoV (Figure 1). Quantification of the DEGs showed that there were 684 genes up‐regulated and 727 genes down‐regulated for the MNV condition, while for the HNoV condition, there were 730 genes up‐regulated and 751 genes down‐regulated (Figure 2a, Dataset S1). Comparison of up‐and down‐regulated genes in the presence of either MNV or HNoV VLPs showed that the viruses share approximately 50% of the DEGs in common (Figure 2b). In addition, we identified 35 genes that were up‐regulated in the presence of one virus but down‐regulated in the presence of the other virus. Thirteen genes were down‐regulated in the presence of MNV but were up‐regulated in the presence of HNoV VLPs, while 22 genes were up‐regulated in the presence of MNV condition but down‐regulated in the presence of HNoV VLPs. Ultimately, these data reveal that, while these related viruses have overlapping effects on *E. cloacae* gene expression, they each result in unique effects that may have distinct downstream implications on bacterial phenotype.

Gene functional enrichment analysis was applied to the significant DEGs (fold change ≥ 2, FDR < 0.05). The top five categories for MNV‐1 and HNoV VLPs contain 81% and 72% of all the DEGs, respectively (Figure 2c). For both viruses, genes associated with the transmembrane are among the most differentially expressed, accounting for approximately 30% of all DEGs for each virus. Both viruses also altered the expression of many genes associated with transport and the cell membrane. For HNoV, there were 103 DEGs (29.2%) in the transmembrane category, 59 (16.7%) in the transport category and 51 (14.4%) in the cell membrane category. For MNV, there were 78 DEGs (29.7%) in the transmembrane category, 42 (16.0%) in the transport category and 33 (12.5%) in the cell membrane category. Further examination of genes within these functional categories revealed a repertoire of genes related to membrane stability. Specifically, *ompR*, *degS*, *nlpI*, *mlaD* and *rcsC* were up‐regulated by one or both viruses, while *ompW*, *rseA*, *degP* and *rpoE* were down‐regulated (Table 1). Changes in the expression of these genes are associated with changes in the production or biogenesis of OMVs in Gram‐negative bacteria (Choi et al., 2017; Nagakubo et al., 2020). Quantitative RT‐PCR validation was also performed on a subset of DEGs, and results showed that trends in gene expression were consistent between RT‐qPCR and RNA‐seq analysis (Figure S2a), although the magnitude of fold change differed for some genes (Figure S2b) as seen in other works (Moriano‐Gutierrez et al., 2019; Wheeler et al., 2019).

**TABLE 1:** *E. cloacae* genes associated with membrane stability and/or OMV formation that are differentially regulated upon interaction with either MNV or HNoV VLPs and their annotated functions (NCBI)

Gene name/locus ID	MNV log2 Fold Change	HNoV log2 Fold Change	Function
*ompF (ECL_02724)*	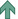 1.6	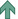 2.3	porin
*ompW (ECL_01652)*	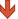 ‐2.1	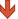 ‐2.0	outer membrane protein
*degS (ECL_04616)*	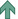 0.7	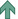 1.0	outer membrane stress sensor serine endopeptidase
*degP (ECL_00965)*	NA	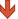 ‐2.2	serine endoprotease
ECL_00220	NA	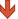 ‐2.3	ompA family lipoprotein
ECL_03245	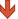 ‐1.6	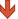 ‐2.5	porin ompC
rcsC (ECL_03522)	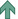 1.2	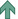 1.2	two‐component system sensor histidine kinase

### 2.2 Phenotypic alterations of the *E. cloacae* outer membrane upon norovirus interaction

**FIGURE 3:**
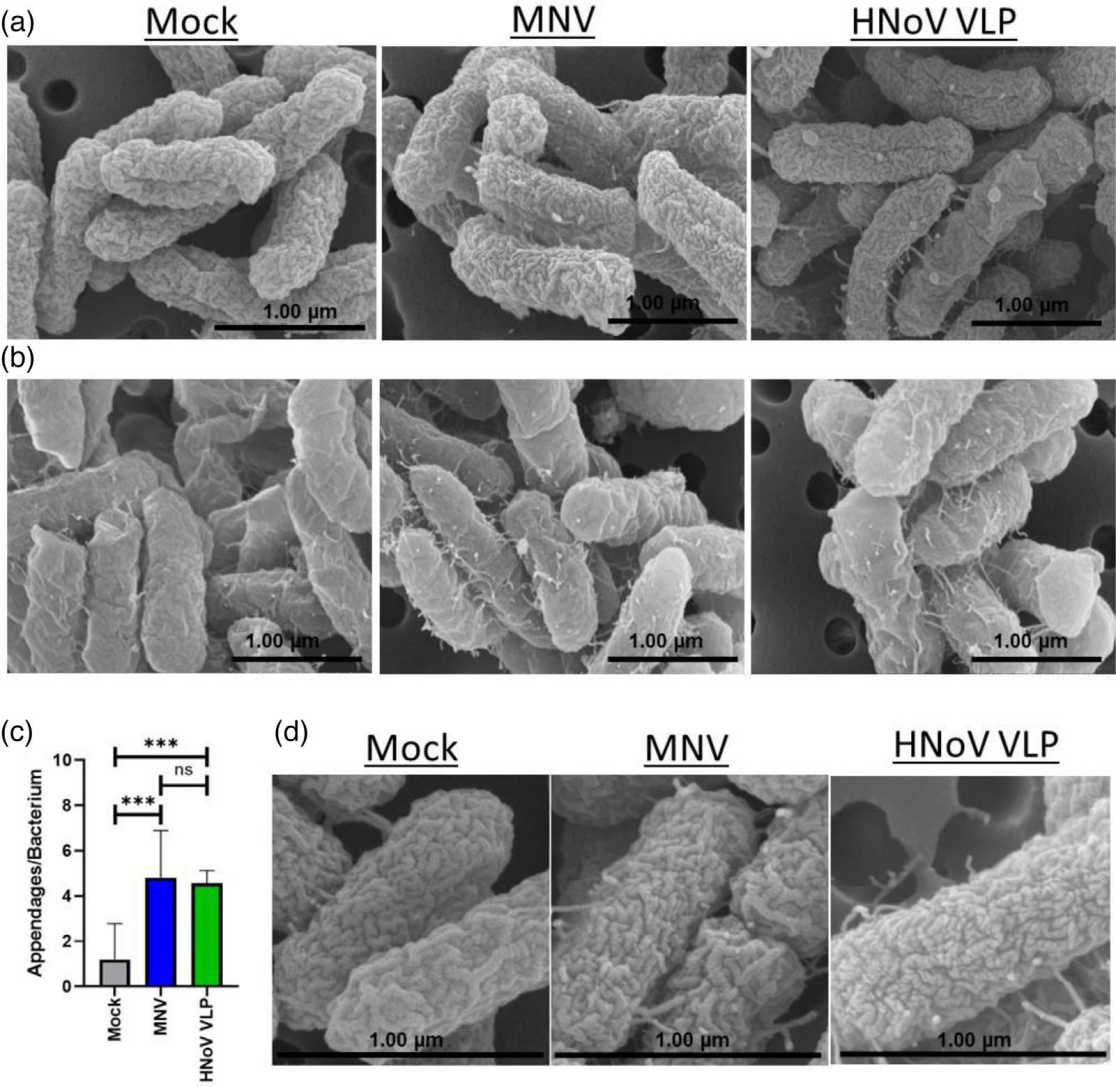
Norovirus interaction alters bacterial surface structures. SEM imaging of *E. cloacae* incubated with mock, MNV or HNoV VLPs, and grown for a) 12 h and b) 1 h. Using SEM images (*n* = 6, *n* = 5 and *n* = 10 for HNoV VLP, MNV and mock, respectively) of on *E. cloacae* after 12 h of exposure to noroviruses c) surface appendages were enumerated at 12 h using SEM images (*n* = 6, *n* = 5 and *n* = 10 for HNoV VLP, MNV and mock, respectively) (***p < 0.001) and d) an increase in surface depressions were observed after 12 h post attachment with viruses

Based on the significant changes in expression of genes associated with membrane stability, we performed a Scanning Electron Microscopy (SEM) analysis *of E. cloacae* incubated in the presence and absence of noroviruses. After attachment, the bacteria were grown for 12 h to allow phenotypes resulting from gene expression changes to develop. Images revealed that incubation with either MNV or HNoV VLPs leads to an increase in bacterial appendages as compared to untreated bacteria, which is prominent at 12 h (Figure 3a) but is observed as early as 1 h post‐viral inoculation (Figure 3b). Quantification of the appendages showed a significant increase in their presence on virus‐exposed bacteria compared to untreated controls (Figure 3c). In addition to the observed increase in bacterial appendages, interaction with noroviruses also resulted in increased ruffling of the bacterial surface after 12 h (Figure 3d). Together, these data demonstrate that interaction with noroviruses induced changes in bacterial surface structures consistent with virally induced changes in gene expression.

### 2.3 Norovirus interaction alters the production of bacterial extracellular vesicles

**FIGURE 4:**
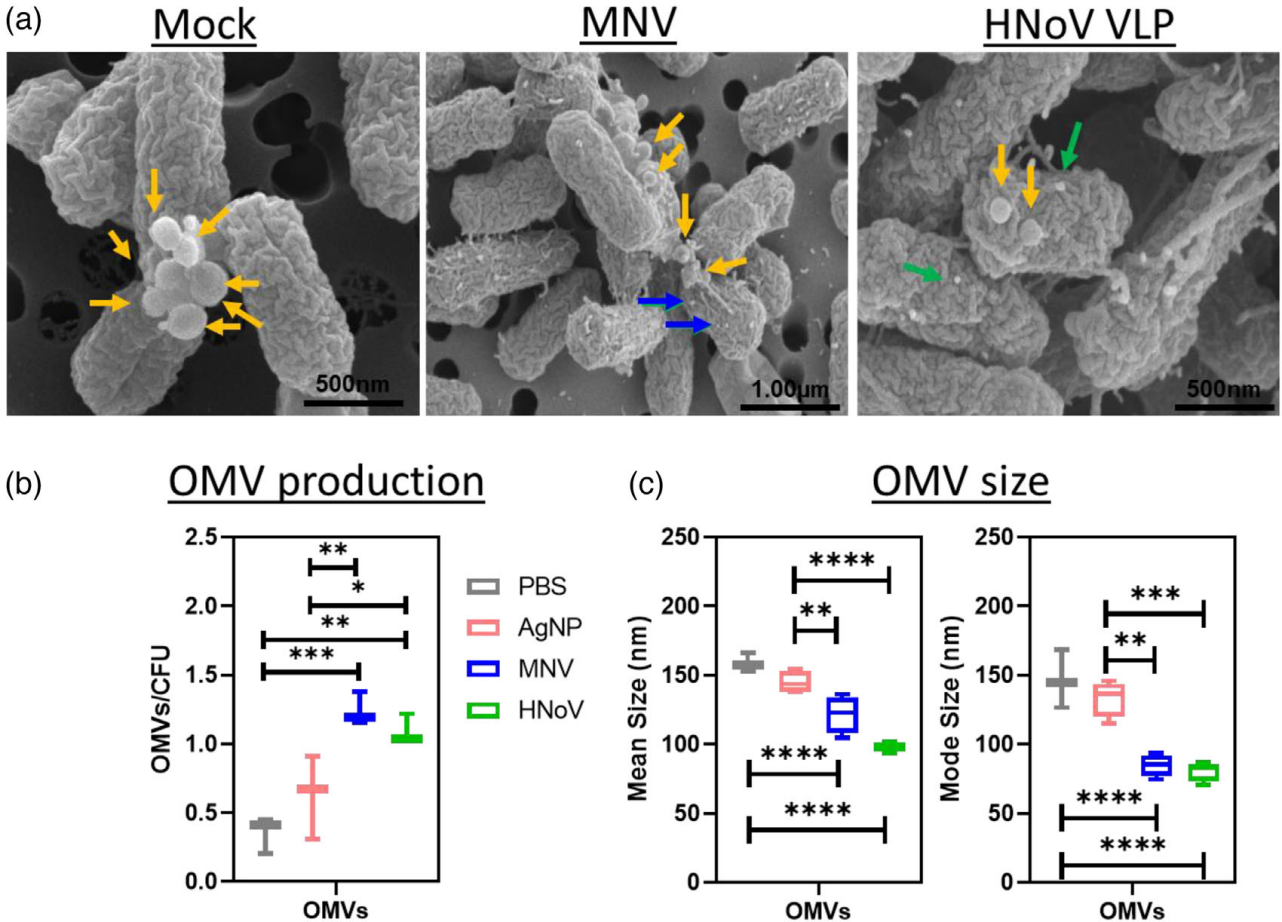
Norovirus interaction alters *E. cloacae* OMV production. *E. cloacae* were incubated in the presence of MNV, HNoV VLPs, PBS or AgNP for 12 h at 37°C to allow OMV formation. a) Scanning electron microscopy (SEM) analysis of bacterial pellets was used to visualize the formation of OMVs in the presence and absence of norovirus. The yellow arrows point to OMVs while blue/green arrows point to viral particles. In addition, NTA was used to determine the, b) quantity of OMV particles/CFU, and c) size (mean diameter and mode diameter) of OMVs produced by *E. cloacae* in the presence and absence of noroviruses. (*p < 0.05, **p < 0.01, ***p < 0.001, ****p < 0.0001)

Based on the changes in expression of genes related to outer membrane stability and surface structure architecture (Figure 2), we examined SEM images for the presence of OMVs (Figure 4a). The extracellular vesicles were obtained using standard ultracentrifugation methods for these specific or similar bacteria (Lee et al., 2018; Pérez‐Cruz et al., 2016), and the quality of the vesicles was assessed by using microscopy for phenotypic analysis and Nanoparticle Tracking Analysis (NTA) to estimate the size range of the vesicles. SEM analysis of bacteria revealed the presence of vesicles in both mock and virus inoculated samples, but a significantly higher number of OMVs were observed in virus‐treated bacteria (Figure S3). To quantitatively assess these observations, NTA of the OMVs was performed. Results showed that incubation with either MNV or HNoV VLPs resulted in a significant increase in the OMV production by *E. cloacae* (Figure 4b). Furthermore, while the mean and mode size of vesicles produced by PBS and AgNP controls did not significantly differ (158.9 and 146.6 nm; 144.95 and 133.58 nm, respectively), OMVs produced in the presence of both human and murine norovirus were significantly smaller compared to the controls, with mean sizes of 98.3 and 121.6 nm, respectively (Figure 4c). Together, these data show that the interaction of bacteria with these viruses results in an increased production of smaller vesicles and further demonstrate that noroviruses induce phenotypic changes, consistent with virally induced changes in gene expression.

**FIGURE 5:**
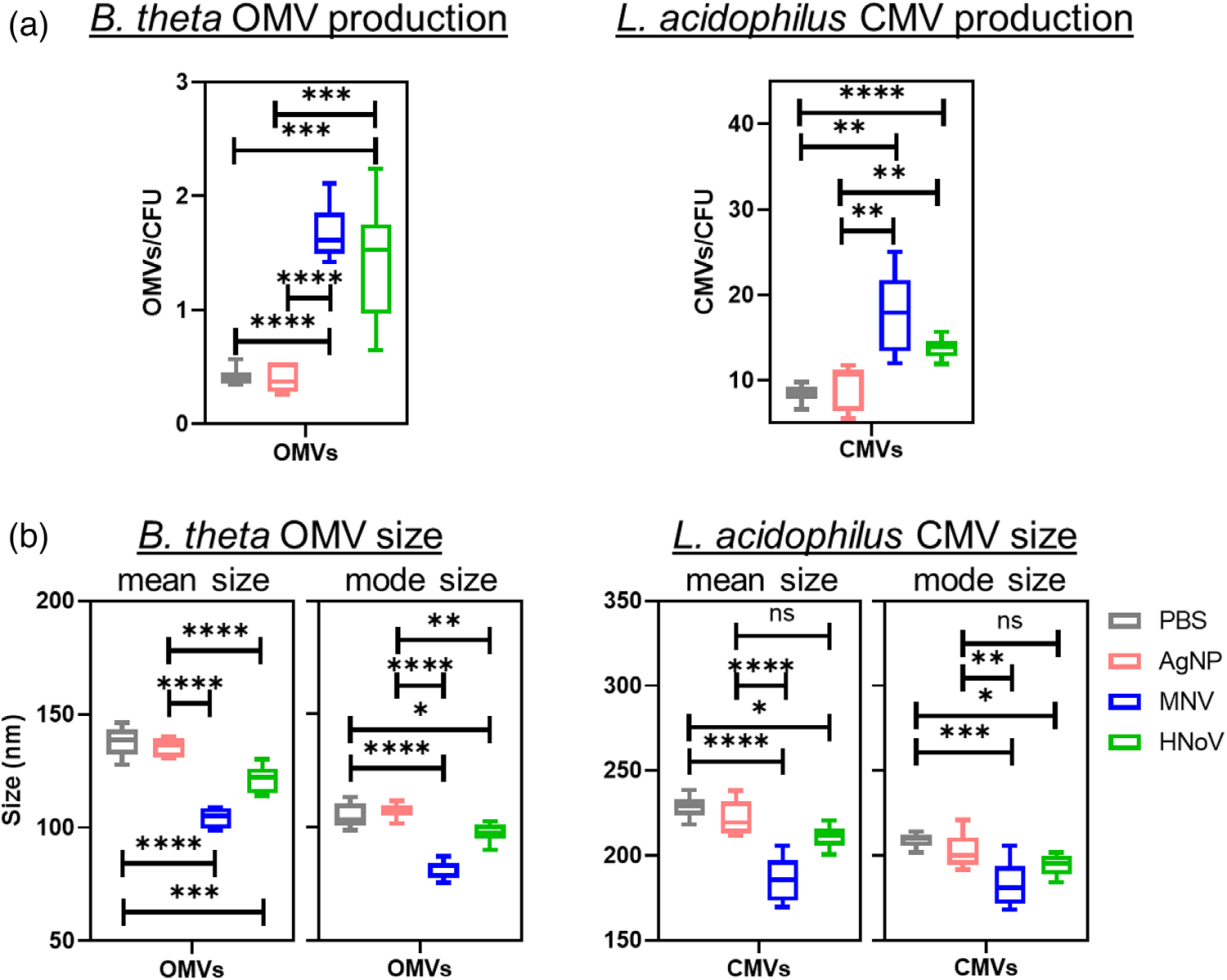
Impact of MNV or HNoV GII.4 VLP exposure on bacterial extracellular vesicle (bEV) production. *B. thetaiotaomicron* (*B. theta*) and *L. acidophilus* were incubated in the presence of MNV, HNoV VLPs, PBS or AgNP; and grown for 12 and 4 h (respectively) at 37°C to allow optimal OMV formation. Nanoparticle tracking analysis was used to determine the quantity and size of a) OMVs produced by *B. theta* and b) cytoplasmic membrane vesicles (CMVs) produced by *L. acidophilus*

While *E. cloacae* is a commensal bacterium commonly found in 40%–80% of the human gut (Sanders & Sanders, 1997), we questioned whether noroviruses also alter bacterial extracellular vesicle production of other bacteria that belong to more predominant phyla of the microbiome. *Bacteroides thetaiotaomicron* (Bacteroidetes phylum) and *Lactobacillus acidophilus* (Firmicutes phylum) were incubated in the presence and absence of MNV and HNoV VLPs, and their extracellular vesicles were analysed using NTA. The impact of viral interaction on membrane vesicle formation by these bacteria was similar to what was observed with *E. cloacae*, where both *B. thetaiotaomicron* and *L. acidophilus* produced significantly more extracellular vesicles in the presence of both viruses compared to either PBS or AgNP controls (Figure 5a). Interestingly, while the Gram‐negative bacteria (*E. cloacae* and *B. thetaiotaomicron*) produced a similar number of vesicles per CFU, the Gram‐positive bacterium (*L. acidophilus*) produced more vesicles than either bacterium generating approximately 13–24x and 10–23x more vesicles, respectively (Figures 4b and 5a). Particularly in the presence of the viruses, Figure 4b shows average OMV/cell for *E. cloacae* of 1.24 (+MNV) and 1.09 (+ HNoV); whereas Figure 5a shows average CMV/cell are observed for *L. acidophilus* of 17.63 (+MNV) and 13.80 (+HNoV), which represent 14x and 13x increases. When comparing vesicles produced by the anaerobic bacteria in the presence of viruses (Figure 5a), the average OMV/cell for *B. thetaiotaomicron* of 1.67 (+MNV) and 1.43 (+HNoV), which represent 10× and 11× increases. In addition, the mean and mode size of OMVs produced by norovirus inoculated *B. thetaiotaomicron* were also significantly smaller than those produced by controls (Figure 5b). However, *L. acidophilus* only produced smaller vesicles in the presence of MNV but not HNoV VLPs compared to AgNP, although vesicles were significantly smaller compared to PBS‐treated samples (Figure 5b). These data indicate that norovirus interactions induce increased vesicle production by a broad range of bacteria. However, the virus‐induced reduction of vesicle size was not universal and may be relegated to Gram‐negative bacterial species. Mechanisms of vesicle biogenesis differ significantly between Gram‐positive and Gram‐negative bacteria, which may also play a role in the ability of noroviruses to induce changes in the vesicle size.

### 2.4 Murine norovirus infection affects bacterial membrane vesicle production in vivo

**FIGURE 6:**
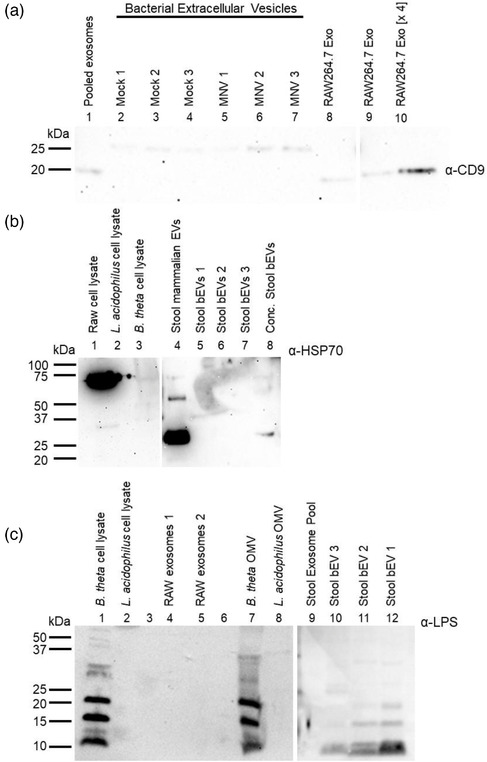
Analysis of bacterial membrane vesicles extracted from murine stool. Stool samples were collected 24 hpi from C57BL/6 mice which were either infected with mock inoculum or MNV. a) Stool derived vesicles were probed with anti‐CD9 antibody. Bands from CD9 tetraspanin are observed in pooled exosomes from depletion steps (Lane 1) as well as from exosomes harvested from RAW 264.7 cells in vitro (Lanes 8–10). Bands were not observed in bEV extracts (Lanes 2–7). b) Exosome depleted vesicles derived from murine stool were also probed with anti‐HSP40 antibody. While RAW 264.7 cell lysates (Lane 1) and stool derived exosomes (Lane 4) were positive for HSP70, bacterial lysates (Lanes 2–3) and stool derived bEVs (Lanes 5–8) were negative for this protein. c) Stool derived vesicles were probed for anti‐LPS antibody. Bands for LPS appeared in the gram‐negative bacterial lysate (Lane 1), in vitro generated OMVs (Lane 7) and in the exosome‐depleted, stool derived bEVs (Lanes 9–12). LPS was not detected in any other sample

**FIGURE 7:**
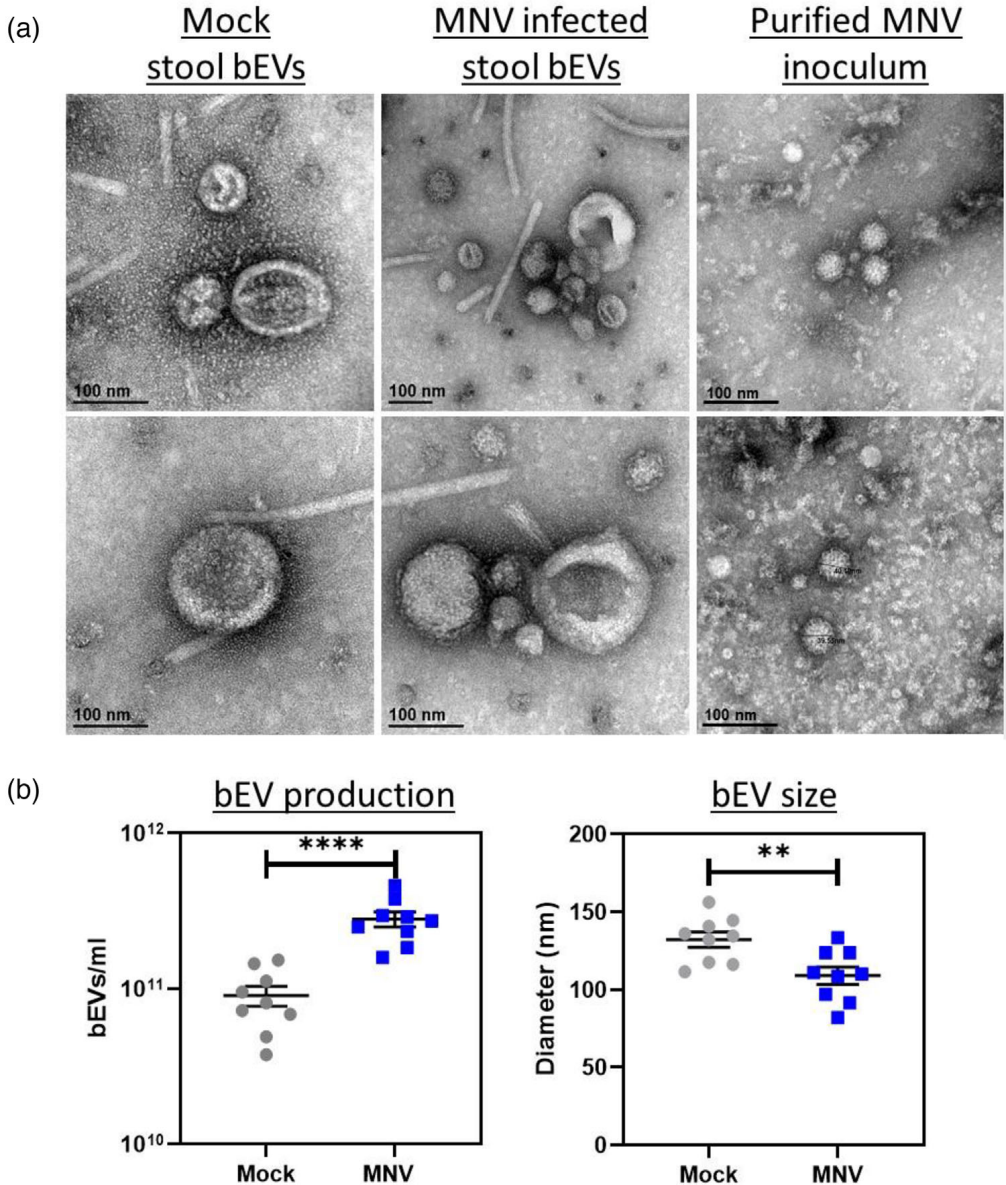
Effect of MNV infection on bacterial extracellular vesicles (bEVs) released in mouse stool. Stool samples were collected 24 hpi from C57BL/6 mice which were either infected with mock inoculum or MNV. a) TEM imaging reveals the presence of stool derived vesicles in both mock and MNV treated stool samples. MNV infected samples contained particles similar in size to particles in purified MNV inoculum. b) Nanoparticle tracking analysis (*n* = 9) determined the quantity (MVs/ml) and mean size (nm) of bacterial bEVs in stool from either mock or MNV infected mouse

**FIGURE 8:**
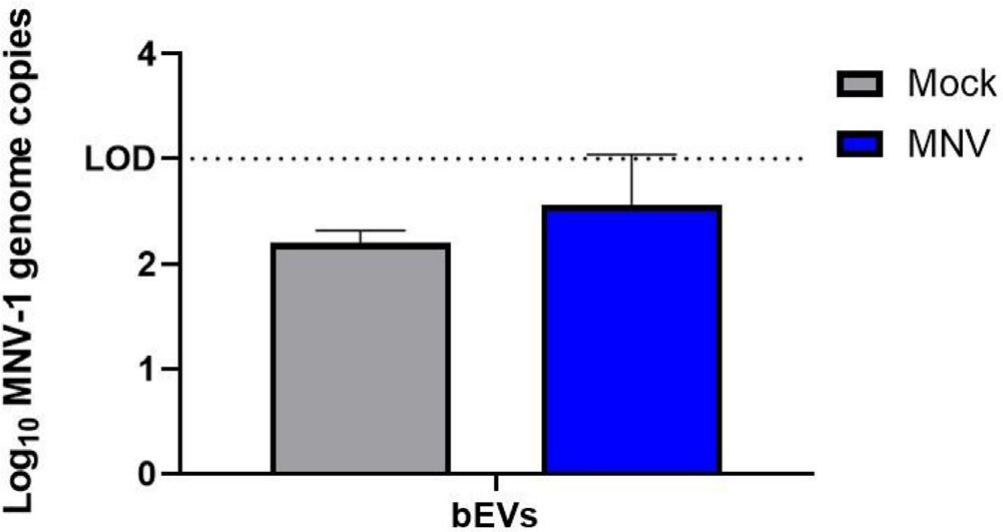
Absence of MNV from stool derived bEVs. Vesicles harvested at 24 hpi from mice infected with either MNV‐1 or mock inoculum were exosome depleted and tested for the presence of virus. RT‐qPCR was performed to detect the presence of the MNV‐1 VP1 gene. All samples generated Cq values below the limit of detection for the assay (*n* = 3)

To determine if these viral effects on vesicle production were biologically relevant, we infected mice with either mock inoculum or MNV‐1. Fecal pellets were collected at 24 h post‐infection (hpi), and extracellular vesicles were isolated and purified from the feces. Anti‐tetraspanin‐coated beads were used to remove host exosomes, where the exosome depletion was confirmed using anti‐CD9 Western blot (Figure 6a). Additional analysis assessing the presence of HSP70 showed all bEV samples were negative for this protein which demonstrates that they do not have detectable contaminants or vesicles from the murine cell membrane (Figure 6b). Furthermore, Western blot showed that after exosome depletion, the vesicles obtained from stool samples were predominantly bacterial in origin due to the presence of LPS in these vesicles, where LPS‐specific bands were absent in the exosomes (Figure 6c). The presence of intact and purified bEVs was confirmed using TEM that revealed vesicles with sizes ranging from 20–250 nm in diameter (Figure 7a). NTA analysis of stool‐derived vesicles showed bEV production was significantly increased in MNV‐infected mice compared to mock‐infected controls (Figure 7b). In addition, the bEVs harvested from MNV‐infected mice were also significantly smaller (Figure 7b). This trend of increased production of smaller vesicles in the presence of MNV is consistent with our in vitro experiments (Figures 4 and 5). Examination of TEM images reveals the presence of smaller vesicles as well. When compared to the TEM of the MNV inoculum, the smaller vesicles approximate the size of the virus (Figure 7a). Thus, to determine if the virus was present in the bEV samples isolated from stool, RT‐pPCR using virus‐specific primers was performed. Results showed that viral genome was not detectable in these vesicles (Figure 8), indicating that the smaller vesicles observed by TEM are not a virus and that the stool‐derived vesicles are devoid of detectable MNV particles. Thus, the significant decreases in bEV size in the stool of MNV infected mice are not due to small MNV particles.

### 2.5 Murine norovirus interaction alters the protein cargo composition of OMVs produced by *E. cloacae*

Shifts in OMV size are linked to changes in the vesicle content (Turner et al., 2018). Based on the reduction in OMV size that occurs in the presence of noroviruses, proteomic analysis was performed on purified OMVs generated by *E. cloacae* in the presence of MNV‐1 or AgNP (denoted as mock) to determine if viral interaction altered the protein cargo of the vesicles. When examining the tandem mass spectra, 441 total proteins were validated using Protein and Peptide Prophet with greater than 95% probability for proteins and peptides, respectively, and at least two identified peptides present per protein (Dataset S2). The identified proteins had varying functions, including transport and signalling, enzymatic activity, membrane assembly, carbohydrate metabolism and fatty acid beta‐oxidation. Interestingly, 181 out of the 441 proteins were found to be of cytoplasmic origin. This presence of cytoplasmic proteins in OMVs is indicative of explosive cell lysis as a mechanism of *E. cloacae* OMV biogenesis, which supports our earlier findings (Bhar et al., 2021). Explosive cell lysis is also indicated by bacterial DNA present within OMVs, and analysis of vesicles produced by *E. cloacae* revealed the presence of genomic DNA, further supporting explosive cell lysis as a mechanism for *E. cloacae* OMV biogenesis (Figure S5a).

**FIGURE 9:**
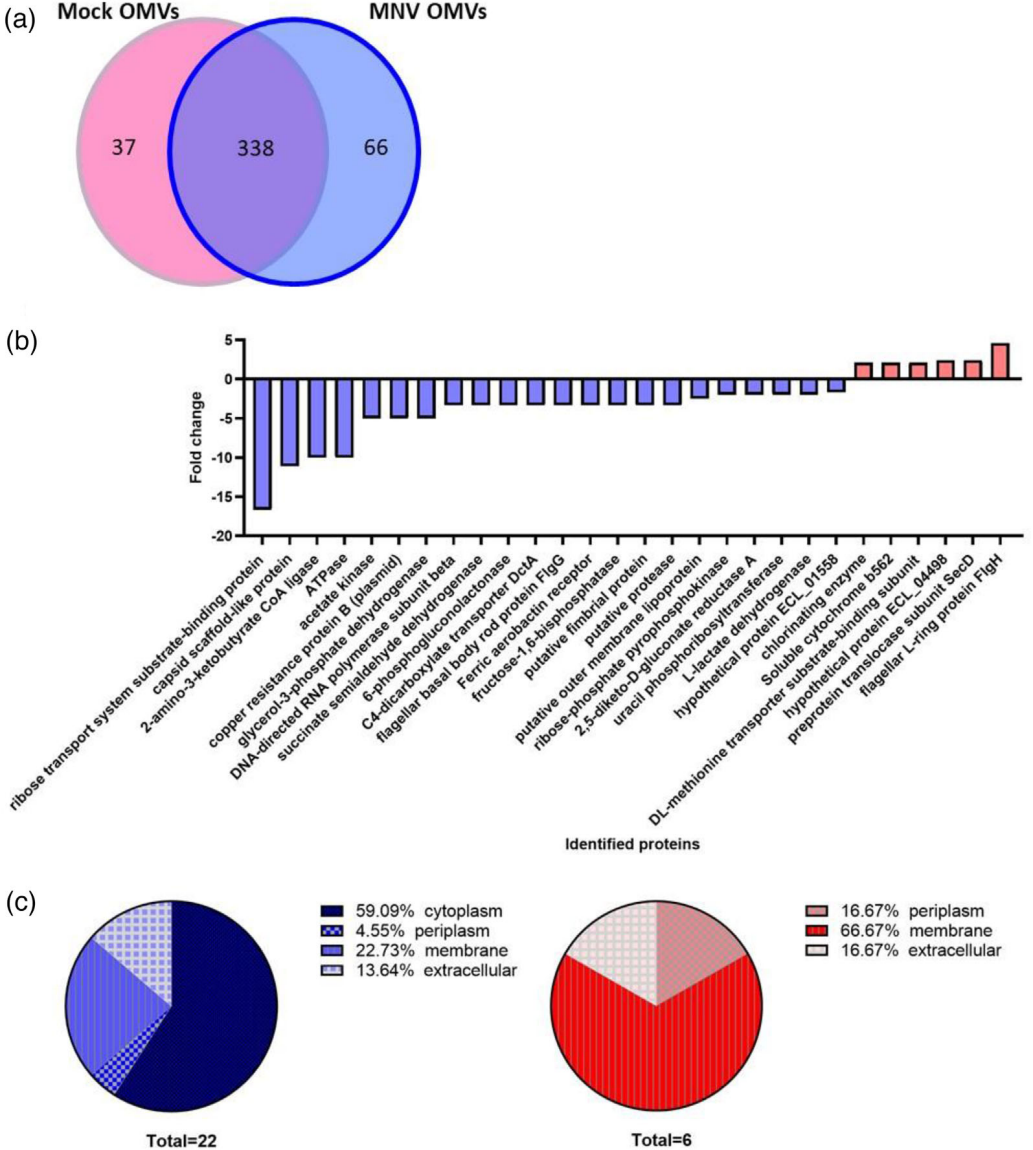
Impact of MNV exposure on the protein content of *E. cloacae* OMVs*. E. cloacae* OMVs were generated in the presence and absence of MNV. After purification, LC/mass spectrometry was used to compare the protein content of the vesicles. Peptide identifications with a probability above 95% and at least two identified peptides were accepted using Scaffold. Protein annotations were done with NCBI GO terms. a) Venn diagram showing the total division of total identified proteins. b) Change in the abundance of proteins found in OMVs generated in the presence of murine norovirus (*p* < 0.05). c) functional categorization of OMV proteins found in increased (red) or decreased (blue) abundance in MNV‐OMVs

When comparing the proteins of OMVs generated in the presence of MNV vs. AgNP, 66 proteins were uniquely present in MNV OMVs, whereas 37 proteins were uniquely present in mock OMVs (Figure 9a). MNV and AgNP OMVs shared 338 proteins in common, and of these proteins found in common, 22 proteins were found to be in lower abundance in MNV OMVs, whereas six proteins were in higher abundance as compared to AgNP OMVs (Figure 9b, Table S1). Most of the proteins (59.09%) with lower abundance in MNV OMVs were of cytoplasmic origin (Figure 9c, blue). On the contrary, proteins having higher abundance in MNV OMVs were mostly membrane‐associated (66.67%; Figure 9c, red). With the availability of the transcriptomic data set, we also explored if these changes in protein abundance correlated with changes in expression of the associated genes. Results showed that MNV interaction decreased expression of genes associated with 4 out of the 22 proteins in lower abundance in MNV OMVs and increased expression of genes that encode for 3 of the 6 MNV OMV higher abundant proteins (Table S2). These observed changes in protein abundance within OMVs also indicated a shift away from explosive cell lysis as the primary mechanism of *E. cloacae* OMV biogenesis. To further explore this shift, genomic DNA in OMVs generated in the presence of MNV was extracted and compared to DNA from mock‐generated OMVs. ImageJ analysis of band intensity revealed that MNV‐OMVs had less DNA than the mock‐OMVs, but the decreased DNA amount was not significant (Figure S5b). Previous analysis of norovirus interaction with *E. cloacae* has shown that, while the viruses bind to most bacteria in the culture, approximately 15% of bacteria are not bound (Madrigal et al., 2020). Given that changes in OMV production may be linked to direct interaction by the virus, unbound bacteria may be producing OMVs via explosive cell lysis, while only virally bound bacteria are shifting away from this mechanism. Therefore, harvesting OMV from a mixed bound/unbound may diminish changes in protein content and may also explain why the decrease in DNA in MNV‐OMVs was not significant. Collectively, these data suggest that MNV binding shifts the mechanism of OMV biogenesis by *E. cloacae* away from explosive cell lysis, and further investigation is needed to confirm these results.

Alterations in OMV protein cargo are known to reflect changes occurring in the original bacterium from which they are derived (Haurat et al., 2011; Nagakubo et al., 2020). Therefore, we next compared the proteomic content of our OMVs to the transcriptomic data of the parental bacteria (Datasets S1 and S2). These comparisons revealed that several proteins which were absent in MNV OMVs were also genetically downregulated in MNV‐exposed *E. cloacae*. Similarly, proteins present only in MNV OMVs had up‐regulated gene expression in MNV‐exposed *E. cloacae* (Tables S2 and S3). This comparison revealed additional differentially expressed genes/proteins that are related to OMV formation. For example, the ATP‐dependent clp protease subunit (Table 2), which plays a role in OMV formation in *E. coli* was identified, where deletion of *clpP* results in increased OMV production (Kulp et al., 2015). In *E. cloacae*, this gene is downregulated in the presence of MNV and is one of the proteins absent in MNV OMVs.

**TABLE 2:** Proteins differentially present in *E. cloacae* MNV OMVs which are also differentially regulated in *E. cloacae* RNA‐seq upon MNV interaction

Absent in MNV OMVs and > 2‐ fold downregulated in *E. cloacae* by MNV			
Identified proteins	Function	Gene Name	Gene Locus ID
Silver binding protein precursor SilE (plasmid)	Stress response	*silE*	ECL _A037
Single‐strand DNA‐binding protein Ssb	DNA replication and repair	*ssb*	ECL_00317
Fructose‐1,6‐bisphosphatase	Gluconeogenesis	*fbp*	ECL_00637
ATP‐dependent Clp protease proteolytic subunit	Hydrolase	*clpP*	ECL_00721
Putative fimbrial protein	Cell adhesion	*fimF*	ECL_01283
Hypothetical protein ECL_00450	Unknown		ECL_00450

Present MNV OMVs only and > 2‐ fold upregulated in *E. cloacae* by MNV			
Identified proteins	Function	Gene Name	Gene Locus ID
Chlorinating enzyme	Biosynthetic process		ECL_03956
Carbamoyl‐phosphate synthase small subunit	Biosynthetic process	*carA*	ECL_00839
ABC transporter arginine‐binding protein	Transport	*artJ*	ECL_02808
Copper resistant protein PcoB	Homeostasis	*pcoB*	ECL_04880
Cytochrome o ubiquinol oxidase subunit II	Oxidoreductase	*cyoA*	ECL_01189
NADH:ubiquinone oxidoreductase, chain C,D	Oxidoreductase	*nuoC, nuoCD, nuoD*	ECL_03630
30S ribosomal protein S10	Translation	*rpsJ*	ECL_04698
50S ribosomal protein L14	Translation	*rplN*	ECL_04687
50S ribosomal protein L6	Translation	*rplF*	ECL_04682
Antimicrobial peptide ABC transporter	Lipid metabolism	*pagP*	ECL_03072

Interestingly, the comparative analysis also revealed increased expression of genes/proteins associated with the induction of bacterial stress. Some examples include chlorinating enzyme, carbomyl phosphate synthase, an ABC transporter arginine binding protein, an antimicrobial peptide ABC transporter, copper resistant protein and oxidoreductases (Table S2). The chlorinating enzyme plays a role in the biosynthesis of polyketide, which has antibiotic and pharmacological properties and can be formed during environmental stress (Doull et al., 1993). In comparison, carbamoyl phosphate synthase is an intermediate enzyme required for both arginine and pyrimidine biosynthesis (Charlier et al., 2013). Interestingly, both arginine and pyrimidine metabolism, along with ABC transporters, were seen to be enhanced under different stress conditions (Murugasu‐Oei et al., 1999; Nagar et al., 2016; Nagayama et al., 2014). In addition, copper‐resistant protein and oxidoreductases are also associated with bacterial stress responses (Anwar et al., 2013; Guest et al., 2017; Quintana et al., 2017; Rensing & Grass, 2003). Collectively, these data demonstrate the correlation between bacterial gene expression and OMV protein content and indicate that norovirus interactions induce a stress‐like condition that may cause the observed hypervesiculation (Figures 4, 5, and 7).

## 3 DISCUSSION

Commensal bacteria dramatically impact enteric viral infection (Baldridge et al., 2015; Jones et al., 2014; Kuss et al., 2011; Robinson et al., 2019; Uchiyama et al., 2014). Likewise, viral infection can also alter the microbiome depending on the virus. For example, norovirus infection is associated with a decrease in Bacteroidetes and Proteobacteria and increases in Firmicutes (Hickman et al., 2014; Patin et al., 2020), while opposite trends are observed during rotavirus infection (Cecil et al., 2019; Engevik et al., 2020; Stentz et al., 2018).

While numerous studies have investigated the large‐scale impact of enteric viruses on the intestinal microbiome, there are no studies to date examining the specific impact these mammalian viruses have on individual bacteria. To address this gap in knowledge, we analysed two distinct but related enteric viruses to determine if interactions of these viruses altered bacterial gene expression. Both murine and human noroviruses were able to induce significant, genome‐wide changes in gene expression in *E. cloacae*, impacting approximately 25% of the bacterial genome. While 50% of the DEGs were shared between the viruses, the viruses also induced a repertoire of genes unique to each pathogen. This is the first known demonstration of mammalian viruses altering the gene expression of a commensal bacterium.

Functional annotation of these DEGs revealed that many of them were linked to transmembrane or outer membrane genes. These DEG categories were notable given that SEM images of virus‐bound bacteria revealed changes in the surface topological features of *E. cloacae*. An increase in the number of bacterial appendages was observed in virally exposed bacteria, and previous research has shown increases in bacterial appendages under conditions of cellular and environmental stress (Ikeda et al., 2020; Yang et al., 2016). We also observed an increase in membrane ruffling upon norovirus interaction with *E. cloacae*, similar to membrane wrinkles observed upon treatment with low concentrations of an antibacterial compound (Schneider et al., 2016). Under these conditions, the wrinkling activity was coordinated with the release of small vesicles, which indicated that stress‐induced changes in the cellular membrane are associated with bEVs production (Schneider et al., 2016). Vesicle production is often linked to stress exposure (Mcbroom & Kuehn, 2007; Turnbull et al., 2016) and can be altered by various stress‐inducing factors (Macdonald & Kuehn, 2013; Mcbroom & Kuehn, 2007; Mcmahon et al., 2012). Based on this, our observed changes in the bacterial surface architecture, and the differential regulation of genes associated with membrane stability and vesical formation, we measured OMV production in the presence and absence of noroviruses. Our studies found that norovirus interaction resulted in higher production of OMVs by *E. cloacae*. Thus, our data collectively indicate that norovirus attachment to *E. cloacae* induces a stress‐like condition in this bacterium.

Given that noroviruses bind to a wide variety of commensal bacteria (Almand et al., 2017; Madrigal et al., 2020), the ability of these viruses to similarly impact other commensals was also examined. Indeed, both *B. thetaiotaomicron* and *L. acidophilus* increased their production of bEVs while exposed to noroviruses, indicating that viral interaction may induce stress responses in many types of commensal bacteria. To determine the biological relevance of these in vitro findings, we quantified bacterial EVs from the stool from MNV infected and uninfected mice. Results showed similar trends to our in vitro work, where increased production of smaller vesicles was found in mice infected with MNV. Therefore, mammalian virus interactions may signal a stress response in commensal bacteria which could have downstream impacts on host responses to viral infection. Future studies examining specific stress responses on individual bacteria as well as the microbiome will provide greater insight into the biological role of these changes on both viral infection and host responses.

Not only did norovirus interactions result in increased bEV production, but the vesicles produced by each of the bacteria tested were smaller when generated in the presence of a virus, indicating that norovirus‐bacteria interactions altered the content of OMVs. Proteomic analysis of OMVs produced by *E. cloacae* revealed differences in protein content in MNV‐OMVs compared to those produced by mock‐inoculated bacteria. Many of these proteins have been previously linked to bacterial stress responses, further supporting that interaction with these viruses induces stress responses. In addition, MNV‐OMVs had a lower abundance of specific proteins, mostly from cytoplasmic origin. Decrease in proteins abundance is correlated to smaller OMV size in *Helicobacter pylori* (Turner et al., 2018), which is linked with changes in OMV migration through epithelial cells, where smaller OMVs prefer entering gut epithelial cells via caveolin‐mediated endocytosis, and larger OMVs enter epithelial cells via endocytosis or micropinocytosis (Turner et al., 2018). While the mode of entry of *E. cloacae* OMVs into host cells is still unknown, virus‐induced changes in vesicle size may alter the cellular entry of these vesicles.

Another implication of the observed proteomic changes in *E. cloacae* OMV content is a shift in the mechanism of vesicle biogenesis. OMVs produced from *E. cloacae* had numerous cytoplasmic proteins and genomic DNA present in their cargo, consistent with what was found in our previous study (Bhar et al., 2021). OMVs produced by explosive cell lysis have cytoplasmic proteins and genomic material in their cargo, while OMVs produced by outer membrane blebbing have periplasmic proteins and outer membrane‐associated proteins only and primarily lack cytoplasmic proteins (Toyofuku et al., 2019; Turnbull et al., 2016). However, when *E. cloacae* vesicles are formed in the presence of MNV, decreases in both cytoplasmic proteins and genomic DNA are observed, indicating a shift away from explosive cell lysis as the mechanism of OMV biogenesis. These vesicles also contain increased periplasmic and membrane‐associated protein, indicating formation through membrane blebbing and suggesting that MNV interaction promotes outer membrane blebbing‐based OMV formation. The biological implications of OMVs produced by these different mechanisms have not yet been explored, but the ability of murine norovirus to induce this shift may provide a platform for evaluating these differences. In addition, as mentioned above, only a portion of the bacterial population is bound by these viruses. Thus, either the virally‐induced changes in the bound bacteria are so dramatic that they are detectable in the mixed population or viral binding induces changes in the bound bacteria that result in production of bacterial products (to include OMVs) that stimulate similar changes in the rest of the population. Future studies will be aimed at determine the role of viral binding on communication within bacterial communities.

Further research is also needed to determine the precise mechanisms responsible for the bacterial ability to sense and respond to eukaryotic viruses. Our gene expression analysis leads to a hypothesis that the bacterial response to these viral particles occurs through one of the bacterial envelope stress response systems, such as the σ^E^ pathway or the two‐component system CpxRA. Broadly speaking, stresses to the outer membrane and misfolded proteins in the periplasm activates the σ^E^ pathway, while stresses to the inner membrane trigger the CpxRA system (Choudhary et al., 2020; Steenhuis et al., 2020). Both envelope stress response systems result in various regulatory functions to different stimuli, which may explain the large number of differentially expressed genes that were observed. Regarding the increased OMV production induced by the presence of noroviruses, the response to misfolded proteins in the periplasm by both the CpxRA and σ^E^ pathways may be related as the accumulation of misfolded proteins in the periplasm is an established route for OMV biogenesis via membrane blebbing. Further support for this is that some of the predicted targets for CpxR and σ^E^ regulation include genes that have previously been found to result in hypervesiculation phenotypes such as *degP*, *degS* and *ompC* (Mcbroom et al., 2006; Schwechheimer & Kuehn, 2015). However, further research into this line of inquiry and how it could relate to the changes we see when norovirus is attached is beyond the scope of this paper and will be a subject for future research.

In conclusion, bacterial extracellular vesicles are produced by both pathogenic and commensal bacteria and serve as a primary mechanism by which bacteria communicate with host intestinal cells (Nagakubo et al., 2020). We have previously observed that commensal bacteria can enhance norovirus infection (Jones et al., 2014), but, in the absence of gross inflammation, they are sequestered to the intestinal lumen and do not travel into the lamina propria where the primary targets of acute norovirus infection, the immune cells, reside (Grau et al., 2017). OMVs, on the other hand, have been shown to easily cross the intestinal epithelium to interact with underlying cells and modulate their immune response, like that of the bacterium from which they are derived (Kaparakis‐Liaskos & Ferrero, 2015; Stentz et al., 2018). Moreover, the mechanism of OMV entry into the cell depends on the size and protein content of OMVs (Turner et al., 2018). As we have found, norovirus interaction with commensal bacteria alters the size and protein content of the OMVs, and we speculate that those OMVs may serve as a mechanism for bacterial enhancement of MNV infection by assisting with viral entry into target cells, modulating the host immune response, or both (Dong et al., 2018). Norovirus interactions with commensal bacteria result in widespread changes in bacterial gene expression, which ultimately gives rise to the increased formation of bEVs. Given the established functions for bEVs, there are potential implications for the role these virus‐induced vesicles may play during viral infection, including altering viral migration across the epithelium and immunological responses to infection. Future in vitro and in vivo studies will aid in elucidating the role of OMVs in viral replication and host responses during infection.

## 4 MATERIALS AND METHODS

### 4.1 Bacterial strains and growth conditions

Bacterial isolates used in this study can be found in Table S4. *E. cloacae* was cultivated in Luria Bertani (LB) medium having 1% NaCl under aerobic conditions at 37°C with constant shaking (220 rpm). *B. thetaiotaomicron* was grown in conditioned Brain and Heart Infusion (BHI) medium supplemented with 0.001% hemin at 37°C under anaerobic conditions using anaerobic chambers and anaerobic gas generator packs (ThermoFisher). *L. acidophilus* was cultivated in De Man, Rogosa and Sharpe (MRS) medium at 37°C under anaerobic conditions.

### 4.2 Murine norovirus production

Plasmid pSPMNV‐1.CW3 (provided by Dr. Stephanie Karst) was used to generate recombinant murine norovirus‐1 (MNV‐1) as previously described (Zhu et al., 2013). Briefly, 293T cells were transfected with five micrograms of pSPMNV‐1.CW3. After 24 h, cells were harvested, lysed, the supernatant collected and centrifuged. RAW264.7 cells were infected with the clarified supernatant (0.05 MOI) and harvested 36–48 hpi. Cells were lysed by freeze‐thaw and the clarified supernatant subjected to ultracentrifugation through 25% sucrose cushion. The virus pellet was resuspended in dPBS. Viral titer was quantified using TCID_50_ assay. Human Norovirus GII.4 VLPs were bought from Creative Biolabs (CBS‐V700). All viral stocks were stored at ‐80°C.

### 4.3 Virus‐bacteria attachment assays

Attachment assays were performed as described (Madrigal et al., 2020). Bacteria were grown until the stationary phase, and the bacterial pellet was washed twice with 1X PBS. The bacterial cell count was adjusted with PBS to a final concentration of 10^8^ cells/ml. Cells were inoculated with either MNV (0.1 MOI), HuNoV VLPs (0.1 μg/ml), silver nanoparticle (AgNP; equivalent volume to that of virus) or PBS. The mixtures were incubated for 1 h at 37°C with constant mixing. These mixtures were then used for OMV purification or processed for RNA extraction. For RNA‐sequencing and qPCR experiments, aliquots of 1 ml were used. For large‐scale attachment assays (200 ml), the total culture volume was concentrated into 5 ml of PBS prior to attachment.

### 4.4 RNA extraction and DNase treatment

RNA*later* (Ambion) was added to the 1 ml aliquots of fresh samples. Extraction was performed using the Zymo RNA MiniPrep kit according to manufacturer's instructions. Following the extraction of the attachment assay samples, gDNA was removed using the Turbo DNA‐*free* kit (Ambion) according to the ‘Rigorous DNase treatment" protocol in the kit instructions.

### 4.5 RNA‐seq

RNA Sample QC, library preparations and sequencing reactions were conducted at GENEWIZ, LLC. 16 RNA samples were quantified using Qubit 2.0 Fluorometer and RNA integrity was checked with 4200 TapeStation. rRNA depletion was performed using Ribozero rRNA Removal Kit (Illumina). RNA sequencing library preparation used NEB Next Ultra RNA Library Prep Kit for Illumina by following the manufacturer's recommendations and library enrichment performed with limited cycle PCR. Sequencing libraries were validated using the Agilent Tapestation 4200, and quantified by using Qubit 2.0 Fluorometer and qPCR. The sequencing libraries were multiplexed and clustered on one lane and loaded on the Illumina HiSeq instrument according to manufacturer's instructions. The samples were sequenced using a 2 × 150 Paired End configuration. Image analysis and base calling were conducted by the HiSeq Control Software. Raw sequence data (.bcl files) generated from Illumina HiSeq was converted into FASTQ files and de‐multiplexed using Illumina's bcl2fastq 2.17 software. One mismatch was allowed for index sequence identification.

### 4.6 Transcriptome analysis

The resulting reads from RNA‐Sequencing were assessed for quality with fastQC (Andrews, 2010) and then adapter removal and trimming was carried out using Cutadapt (Martin, 2011). Quality controlled reads were aligned to the *E. cloacae* genome (NCBI) using Bowtie2 (Langmead & Salzberg, 2012). Counts were assigned to genes by featureCounts (Liao et al., 2014). Normalisation was carried out by the cqn R package (conditional quantile normalisation) to remove GC‐content and length biases (Hansen et al., 2012). The normalisation offsets were applied to the raw counts for differential gene analysis in the DESeq2 program as recommended by the DESeq2 manual (Love et al., 2014). DESeq2 uses a negative binomial generalised linear model and an internal normalisation that accounts for sequencing depth. Samples were compared pairwise by looking at the expression levels of MNV, HNoV VLPs and silver nanoparticle conditions versus the PBS control condition. PBS was chosen as our control condition as it serves as a baseline without any additional viral or silver particles added to the sample. Genes with less than 10 total raw counts were removed prior to running the DESeq2 program and additional independent filtering parameters were kept at DESeq2's defaults. The DESeq2 package uses a Wald test to determine the statistical significance of the differential expression results and the Benjamini‐Hochberg correction is used to correct for multiple testing. The adjusted *p*‐value found from the Benjamini‐Hochberg correction was used for our cut‐off of < 0.05. A full list of fold changes and adjusted p‐values after DESeq2 can be found in Dataset S1. Volcano plots were created using the EnhancedVolcano R package and use an adjusted *p*‐value threshold of *p* < 0.05 and a log_2_ fold change cut off of ±1 (Blighe et al., 2020). Functional annotation was conducted by use of Uniprot Knowledgebase (UniProtKB) keywords via the Database for Annotation, Visualisation and Integrated Discovery (DAVID) v6.8 annotation tool (Huang et al., 2009a, 2009b; T. UniProt Consortium 2018).

### 4.7 RT‐qPCR

The list of primers is provided in Table S5. For validation of RNA‐seq results, RNA was DNase treated (Ambion) and cDNA generated using Promega M‐MLV. PowerUp SYBR Green Master Mix (Applied Biosystems) 300 nM of primers as used to amplify cDNA on a QuantStudio 3. *rpoB* and *groL* were used as endogenous controls. RT‐negative controls were included for all samples. Relative gene expression was calculated using the 2∧^−ΔΔCT^ method by normalising the target gene expression to the geometric mean of *rpoB* and *groL*, then normalising the samples to the PBS control.

### 4.8 Production, isolation and purification of EVs

Virus‐bacteria attachment assays were performed as described above through the 1 h incubation at 37°C. Following this step virus:bacteria cultures were inoculated into 200 ml of LB (*E. cloacae*), conditioned BHI (*B. thetaiotaomicron*) or MRS (*L. acidophilus*) and grown at 37°C for 12, 12, or 4 h, respectively to achieve maximum EV production. OMVs from *E. cloacae* and *B. thetaiotaomicron* were harvested as previously described (Bhar et al., 2021). Clarified supernatants of the culture were ultracentrifuged at 25,000 x g for 20 min, followed by filtration and centrifugation at 150,000 x g for 2 h. OMV pellets were resuspended in dPBS and ultracentrifuged at 150,000 x g. To obtain purified CMVs from *L. acidophilus*, cultures were centrifuged at 3200 x g for 20 min at 4°C. The clarified supernatant was ultracentrifuged at 25,000 x g for 20 min at 4°C followed by filtration through 0.22 μm filter. The final supernatant was ultra‐centrifuged at 175,000 x g for 2 h at 4°C. The CMVs were resuspended in dPBS and centrifuged at 175,000 x g for 2 h at 4°C. The OMV or CMV pellets was resuspended in 500‐μl dPBS supplemented with protease inhibitor cocktail (Thermo Fisher #A32955) and stored at 4°C for use within 2 weeks or at ‐80°C for later applications.

### 4.9 Purification of bacterial EVs from stool of mice

C57BL/6 mice were infected with 10^7^ MNV‐1 or mock inoculum and stool sample were collected at 24 hpi. Twenty‐five milligrams of feces were homogenised in 500 μl PBS and centrifuged. The supernatant was transferred to a new tube and the fecal pellet resuspended in 500 μl PBS. Homogenisation, centrifugation and supernatant transfer were repeated for three times to obtain 2 ml of supernatant. The supernatant was centrifuged at 10,000 × g for 30 min to pellet bacteria, larger particles, and large EVs. This clarified supernatant was filtered through 0.22 μm filter and ultracentrifuged at 110,000 × g for 2 h at 4°C in Beckman Optima XE‐90. The supernatant was removed and the pellet containing extracellular vesicles (EVs) were resuspended in 4 ml PBS and ultracentrifuged at 110,000 × g for 70 min at 4°C. The resuspension and ultracentrifugation were repeated three times to wash away any stool contaminants. The final pellet was resuspended in 500 μl dPBS with protease inhibitor cocktail.

Murine exosomes were removed from the vesicle suspension using the Pan Exosome Isolation Kit (Miltenyi Biotech #130‐117‐039) following the manufacturer's instructions. Microbeads were incubated with the vesicle solution and the mixture loaded onto a μMACS column on a magnetic field of μMACSTM separator. The exosomes were retained on the magnetic column while flow through solution containing bacterial EVs was collected and analysed by negative staining and NTA.

### 4.10 Western Blot

Exosomes and stool‐derived bacterial extracellular vesicles were lysed with RIPA buffer then separated by SDS–PAGE on a 4%–12% Bis‐Tris acrylamide gel with XT‐MES running buffer. The separated proteins were then transferred onto a PVDF membrane. The membrane was blocked with 5% nonfat milk followed by incubation with primary and then with secondary antibodies (CD9‐ 1:1000 and 1:10,000 dilutions, System Bioscience # EXOAB‐KIT‐1; HSP70‐ 1:1000 dilution, Invitrogen #MA3‐006; Lipid A LPS‐ 1:1000 dilution, Invitrogen #PA1‐73178;). The detection of the signal was done as previously described (Hui et al., 2021).

### 4.11 Electron microscopy

Scanning electron microscopy (SEM) was performed as described previously (Bhar et al., 2021). Briefly, bacterial pellets were fixed with Trump's fixative, washed with 0.1M sodium cacodylate, pH 7.24 and then fixed with OsO4. The sample is dehydrated in 25%–100% graded ethanol series, dried by Tousimis Autosamdri‐815 and mounted on 12 mm Carbon Conductive Adhesive Tab and aluminum stub with sputter gold/palladium coating before being imaged by Hitachi SU‐5000 FE‐SEM. Negative staining was performed using Transmission electron microscopy (TEM) as described previously (Bhar et al., 2021). Briefly, purified bacterial OMVs pellet, stool derived bEVs or concentrated MNV‐1 were resuspended in fixating solution and 10 μl droplet of the homogenate was poured onto 400‐mesh carbon coated Formvar nickel grid. The excess solution was removed, and the grid was fixed. The grid was stained using floatation on 10 ml of 1% aqueous uranyl acetate and observed with FEI Tecnai G2 Spirit Twin TEM. Finally, images were obtained using Digital Micrograph software and a Gatan Ultrascan 2kX camera.

### 4.12 Nanoparticle tracking analysis (NTA)

bEVs were diluted (1:300 for *E. cloacae* bEVs and 1:500 times for *B. thetaiotaomicron* and *L. acidophilus* bEVs) in filtered autoclaved water and loaded onto NanoSight NS300 sizer and counter (Malvern NanoSight NS300)(10). The particle size and quantity was recorded for 60 s per technical replicate, with five technical replicates for each sample and at least three biological replicates (*n* = 9 for mouse stool derived bEVs).

### 4.13 In‐gel trypsin digestion

OMV proteins were purified and precipitated using TCA protein precipitation assay. The final sample in Bolt LDS sample buffer and Bolt sample reducing agent was heated at 70°C for 10 min. Samples were run through 30% of the length of 4%–12% Bis‐Tris SDS PAGE gel and stained using GelCode Blue. Sample lanes were cut into 1–2 mm cubes, and in‐gel tryptic digestion was performed as described previously (Bhar et al., 2021; Hui et al., 2021). The final peptide solution was lyophilized and analysed by mass spectrophotometry.

### 4.14 Proteomic analysis

Ultrahigh‐Performance Liquid Chromatography (UHPLC) coupled to Orbitrap Fusion mass spectrometer (Thermo Scientific) was used to analyse the protein content of OMV samples described above. Liquid chromatography was performed in Thermo EASY nano‐LC, which was directly interfaced with Orbitrap Fusion MS, where the scan rate was 350–1800 m/z and data were acquired at 120K resolution. Tandem mass spectra were extracted, charge state deconvoluted and deisotoped by Proteome Discoverer version 2.4.1.15. All MS/MS samples were analysed using Sequest (Thermo Fisher Scientific, version IseNode in Proteome Discoverer 2.4.1.15) and in addition, the X! Tandem [The GPM, thegpm.org; version X! Tandem Alanine (2017.2.1.4)]. Sequest was set up to search Uniprot database (5527 entries) assuming the digestion enzyme trypsin. X! Tandem was set up to search a reverse concatenated database (11,268 entries), also assuming trypsin. Sequest and X! Tandem were searched with a fragment ion mass tolerance of 0.60 Da and a parent ion tolerance of 10.0 PPM. Carbamidomethyl of cysteine was specified in Sequest and X! Tandem as a fixed modification. Met‐loss of methionine, glu → pyro‐Glu of the N‐terminus, ammonia‐loss of the N‐terminus, gln → pyro‐Glu of the n‐terminus, oxidation of methionine and acetyl of the N‐terminus were specified in X! Tandem as variable modifications. Met‐loss of methionine, met‐loss+Acetyl of methionine, oxidation of methionine and acetyl of the N‐terminus were specified in Sequest as variable modifications.

Scaffold (version 4.11.0, Proteome Software Inc.) was used to validate MS/MS based peptide and protein identifications. Protein identifications were accepted if they could be established at greater than 95.0% probability and contained a minimum of two identified peptides by the Scaffold Local FDR algorithm with a decoy FDR < 5%. Protein probabilities (95%) were assigned by the Protein Prophet algorithm (Nesvizhskii et al., 2003). Proteins that contained similar peptides and could not be differentiated based on MS/MS analysis alone were grouped to satisfy the principles of parsimony. The samples' quantification was performed using a weighted spectral count, and the ratio was calculated between the treatment and control samples, where a minimum value of 0.5 was used. The student's *t*‐test was used to assign statistical significance to the comparisons (*p* > 0.05) and proteins having greater or lesser than 1.5 fold change were analysed. In addition, proteins were annotated with GO terms from NCBI (Ashburner et al., 2000) (downloaded Nov 13, 2020).

Uniprot (http://www.uniprot.org/) obtained the protein sequences from the database of *E. cloacae* subsp. *cloacae* ATCC 13047(10). The functions of OMV proteins were acquired from Uniprot, InterPro (https://www.ebi.ac.uk/interpro/), Pfam (https://pfam.xfam.org/) and Kegg (https://www.genome.jp/kegg/). UniProt denoted the localisation of most proteins or the functions were predicted by Cello2go (http://cello.life.nctu.edu.tw/cello2go/) and psortb (https://www.psort.org/psortb/).

### 4.15 Purification of genomic DNA from OMVs

Equal particle count of OMVs (10^13^) were treated with 2U DNase (Ambion). Wizard Genomic DNA isolation kit (Promega) was used to purify DNA using the manufacturer's instruction for Gram‐negative bacteria. DNA was loaded to 1% agarose gel and run at 100V for 30 min. The gel was visualized under UV and imaged. ImageJ was used to obtain the mean grey value (band intensity).

### 4.16 Statistical analysis

One‐way ANOVA was used to analyse NTA results. Statistical significance of the qPCR graphs was measured using two‐way ANOVA with Bonferroni post‐test.

## AUTHOR CONTRIBUTIONS

Conceptualization: M.K.J; Methodology: M.K.J, M.B.P. and M.J.E.; Data Curation: C.A.M. and S.B.; Formal analysis: C.A.M., S.B., M.J.E., M.K.J; Supervision: M.K.J. and M.J.E.; Validation: S.B., C.A.M. and M.J.E; Investigation: S.B., C.A.M., M.B.P., M.K.J., M.J.E; Visualization: S.B., C.A.M., M.K.J; Resources: M.K.J. and M.J.E; Writing: S.B., C.A.M., M.K.J., M.J.E.; Project administration: M.K.J. and M.J.E; Funding acquisition: M.K.J. and M.J.E.

## Supporting information

DatasetS1

DatasetS2

Supporting information
