# Supplementary material for: Interaction with mammalian enteric viruses alters outer membrane vesicle production and content by commensal bacteria
Source: J Extracell Vesicles. 2022 Jan 4;11(1):e12172. doi: 10.1002/jev2.12172 (PMC8725172; doi:10.1002/jev2.12172)
Supplement: Supplementary file 3 — Supporting information [file JEV2-11-e12172-s002.pdf]

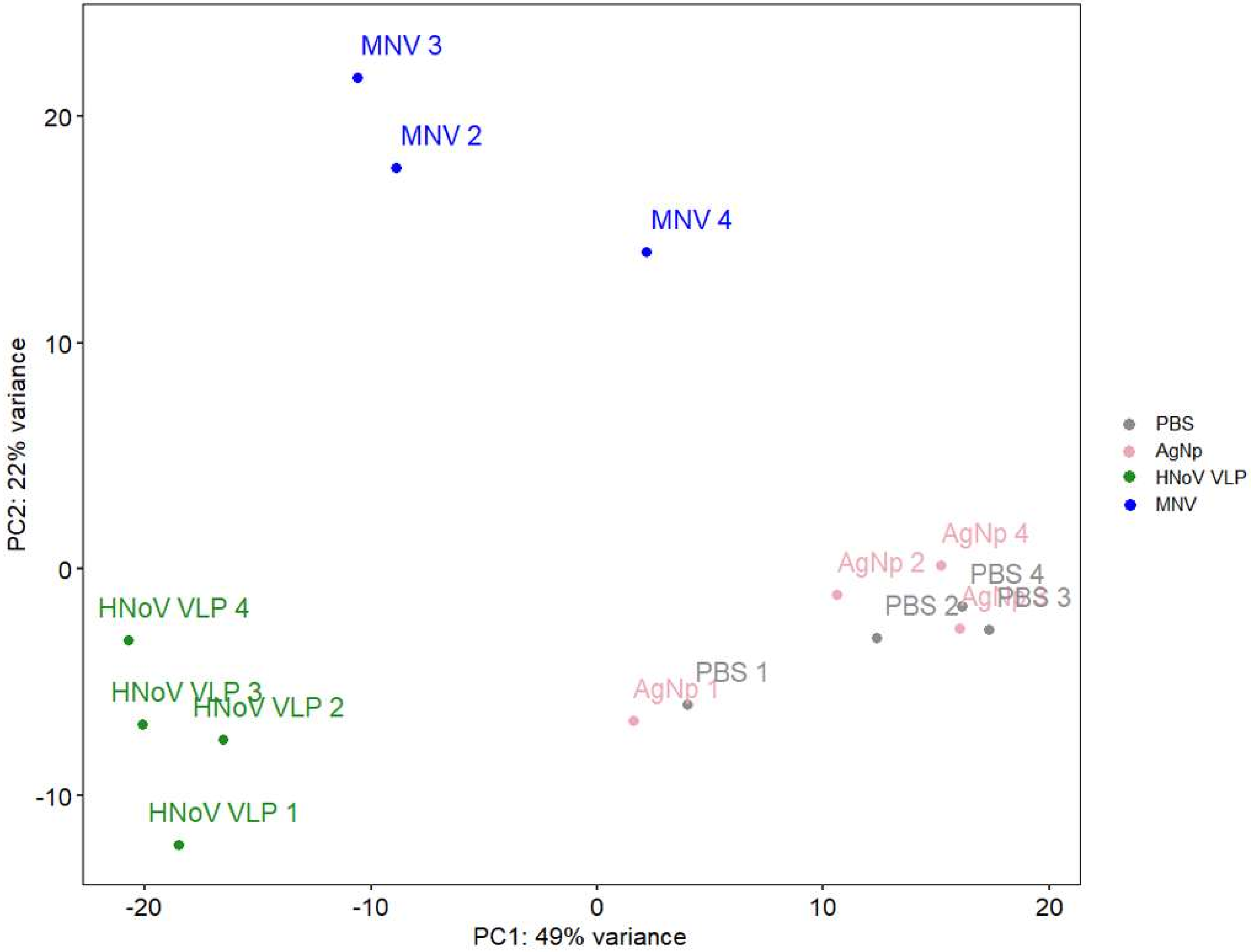

Figure S1. Differential gene expression by *E. cloacae* clusters based on virus exposure.. Cultures of *E. cloacae* (10^8 CFU/mL) were incubated with MNV-1.CW3 (MOI = 1.0), HNoV GII.4 VLPs (0.1 mg/mL), AgNP (equivalent amount to HNoV) or PBS for 1 hr at 37°C. Principal component analysis of the transcriptional profiles of E. cloacae cells show distinct grouping of the mock cells (PBS (n = 4) and AgNp (n = 4)), HNoV-attached cells (n = 4), and MNV-attached cells (n = 3). Raw data of RNA-seq was normalized using the cqn (conditional quantile normalization) and DESeq2 R packages, then rlog transformed.

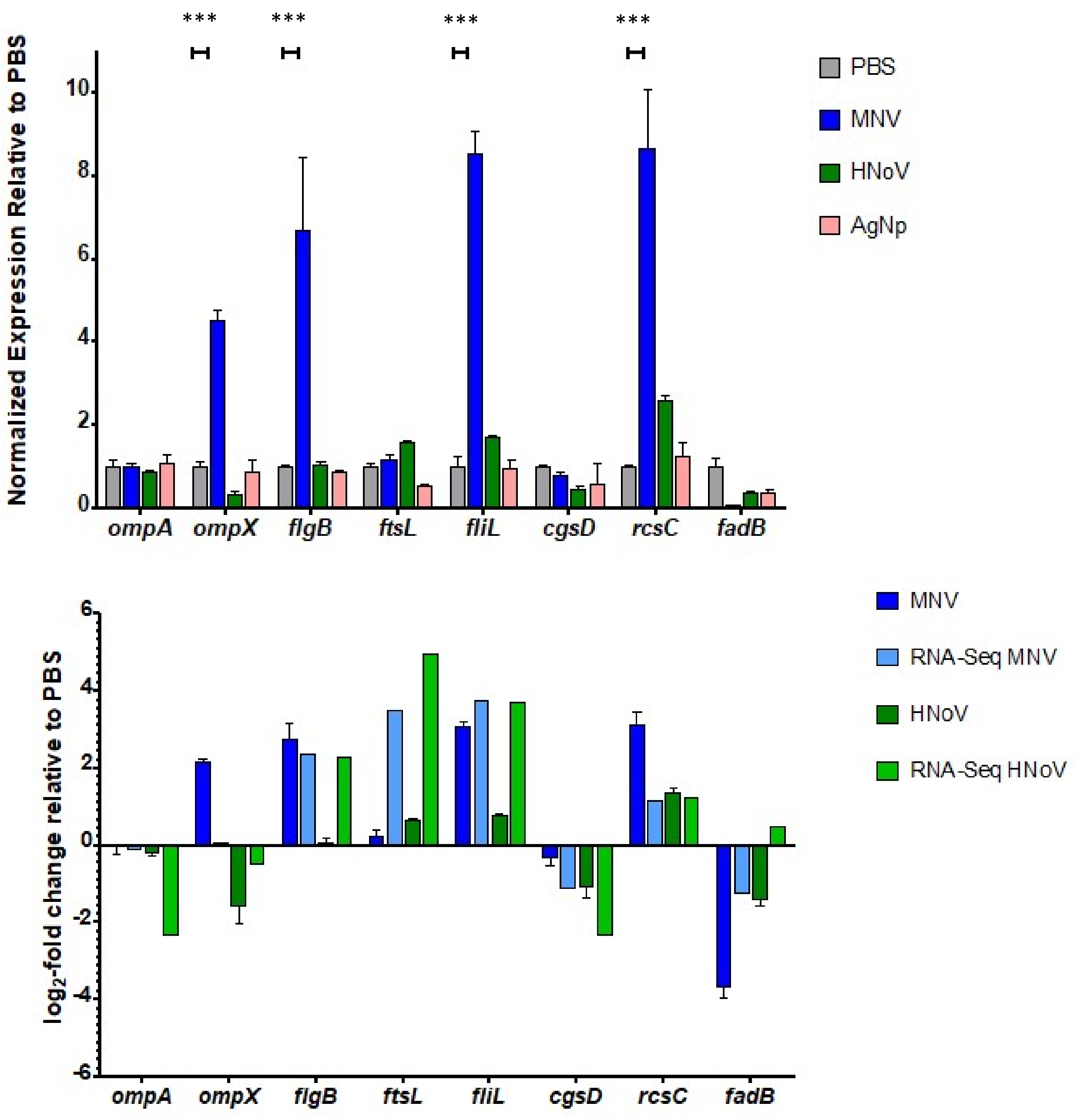

Figure S2. RT-qPCR validation of select genes differentially expressed in RNA-seq analysis. Log2 normalized expression of these genes in RNA seq analyses was compared to qPCR expression analysis of *E. cloacae* after incubation with murine norovirus and human norovirus VLPS (n=3). The results show correlation (spearman coefficient of correlation = 0.762, p = 0.028 for MNV, and 0.619, p = 0.09 for HNoV) between the RNA-sequencing and the qPCR. AgNp condition is not shown as expression of these genes was not significantly altered in the RNA-sequencing analysis.

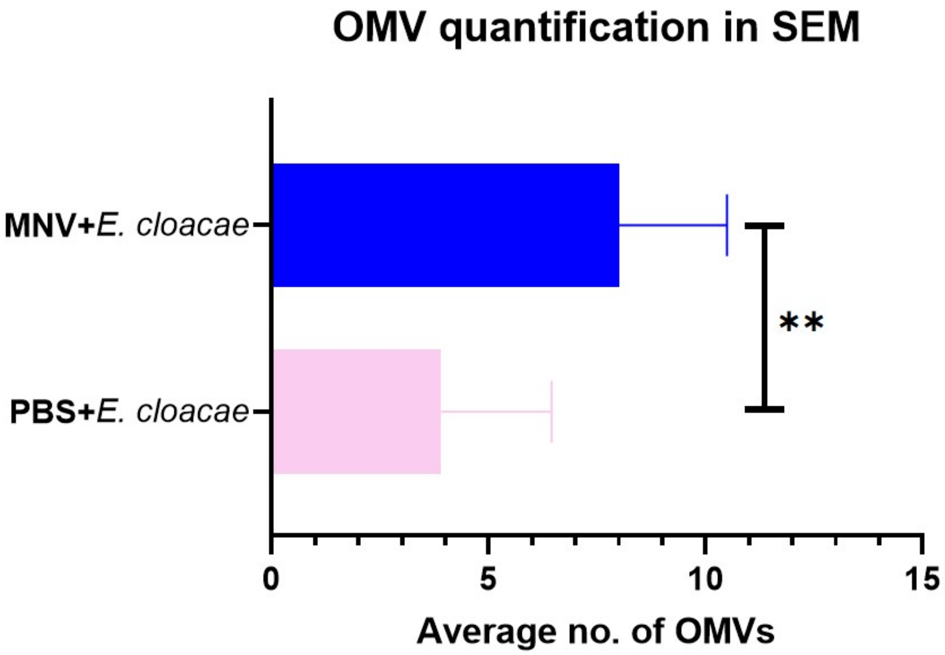

Figure. S3. *E. cloacae* produces more OMVs upon norovirus attachment. SEM analysis shows that MNV attachment leads to about 2-fold OMV release from *E. cloacae.* (n=10 frames each PBS and MNV)

a

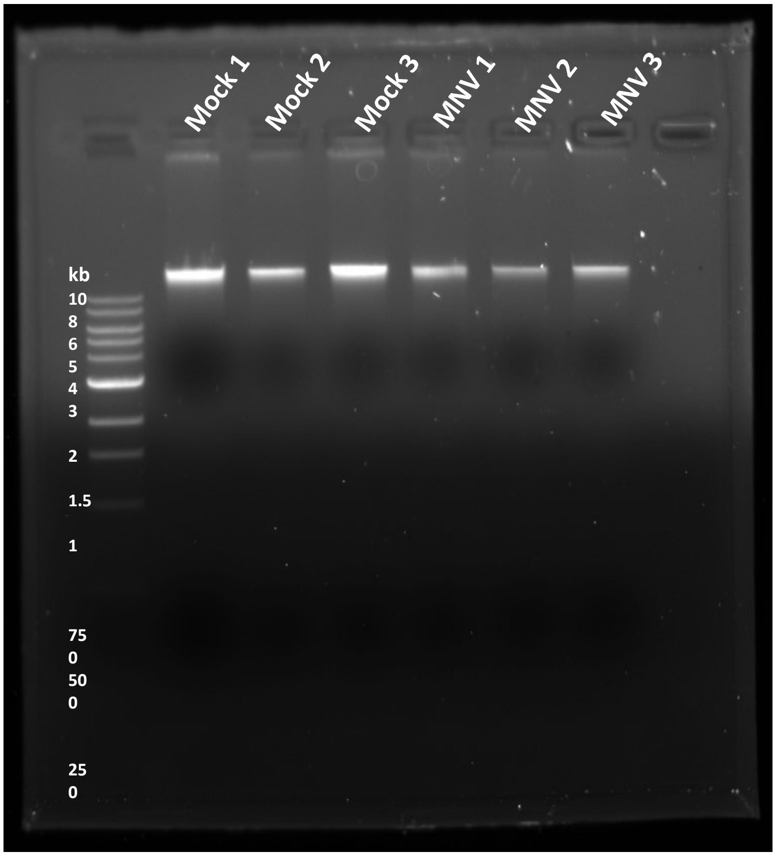

b

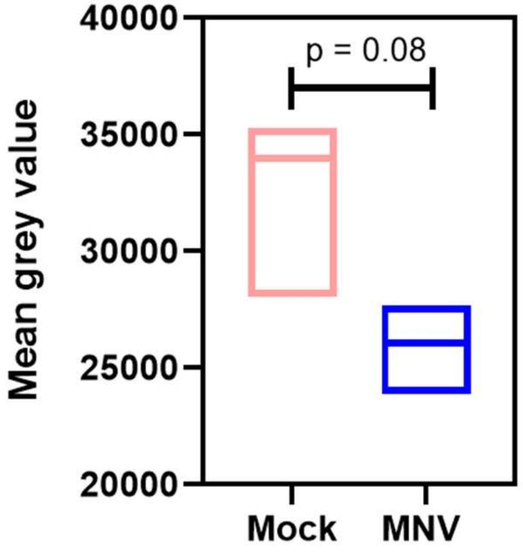

Figure S4. *E. cloacae* OMVs generated in the presence and absence of MNV contain DNA. **a.** A 1% agarose gel shows the presence of genomic DNA in OMVs derived from AgNP treated *E. cloacae* (mock) and MNV treated *E. cloacae* (MNV). **b.** ImageJ software was used to quantify the band intensity of the genomic DNA present in mock OMVs and MNV OMVs. (n=3)

Table S1. Differentially abundant OMV proteins.

Identified Proteins	Accession	T-Test p-value	Fold Change by Category	Function	Location
ribose transport system substrate-binding protein	YP_003615601.1	0.00083	-16.6667	transport	periplasm
capsid scaffold-like protein	YP_003614903.1	< 0.00010	-11.1111	phage protein	cytoplasm
2-amino-3-ketobutyrate CoA ligase	YP_003610658.1	0.0002	-10.0000	biosynthesis	cytoplasm
ATPase	YP_003614179.1	0.012	-10.0000	chaperone	cytoplasm
acetate kinase	YP_003614123.1	0.0023	-5.0000	biosynthesis	cytoplasm
copper resistance protein B (plasmid)	YP_003602514.1	0.0023	-5.0000	homeostasis	outer membrane
glycerol-3-phosphate dehydrogenase	YP_003615265.1	0.0039	-5.0000	metabolism	cytoplasm
DNA-directed RNA polymerase subunit beta	YP_003610761.1	0.003	-3.3333	transcription	cytoplasm
succinate semialdehyde dehydrogenase	YP_003615099.1	0.012	-3.3333	catabolism	cytoplasm
6-phosphogluconolactonase	YP_003613455.1	0.012	-3.3333	glucose metabolism, pentose phosphate shunt	cytoplasm
C4-dicarboxylate transporter DctA	YP_003615405.1	0.036	-3.3333	transport	inner membrane
flagellar basal body rod protein FlgG	YP_003613052.1	0.039	-3.3333	cell motility	extracellular
Ferric aerobactin receptor	YP_003613803.1	0.039	-3.3333	homeostasis	cell outer membrane
fructose-1,6-bisphosphatase	YP_003611152.1	0.039	-3.3333	gluconeogenesis	cytoplasm
putative fimbrial protein	YP_003611793.1	0.039	-3.3333	Cell adhesion	extracellular
putative protease	YP_003612209.1	0.048	-3.3333	hydrolase	cytoplasm

putative outer membrane lipoprotein	YP_003614419.1	0.029	-2.5000	transport, pathogenesis	cell outer membrane
ribose-phosphate pyrophosphokinase	YP_003612101.1	0.00073	-2.0000	biosynthesis	cytoplasm
2,5-diketo-D-gluconate reductase A	YP_003614813.1	0.0054	-2.0000	biosynthesis	cytoplasm
uracil phosphoribosyltransferase	YP_003614271.1	0.0054	-2.0000	metabolism	cytoplasm
L-lactate dehydrogenase	YP_003610677.1	0.018	-2.0000	oxidoreductase	cytoplasmic membrane
hypothetical protein ECL_01558	YP_003612066.1	0.037	-1.6667	phage protein	extracellular
chlorinating enzyme	YP_003614439.1	0.0061	2.1000	biosynthesis	extracellular
Soluble cytochrome b562	YP_003612788.1	0.0078	2.1000	electron transport	periplasm
DL-methionine transporter substrate-binding subunit	YP_003611513.1	0.05	2.1000	transport	cytoplasmic membrane
hypothetical protein ECL_04498	YP_003614976.1	0.032	2.4000	stress response	membrane
preprotein translocase subunit SecD	YP_003611675.1	0.035	2.4000	transport	cytoplasmic membrane
flagellar L-ring protein FlgH	YP_003613051.1	0.017	4.6000	motor activity	cell outer membrane

Table S2. Differentially abundant OMV proteins whose genes were differentially expressed in *E. cloacae* upon norovirus interaction.

Differentially Abundant Proteins			
**MNV Upregulated by at least 1 log_2 fold change**			
**Identified Proteins**	**Function**	**Gene Name**	**Gene Locus ID**
chlorinating enzyme	biosynthesis		ECL_03956
Soluble cytochrome b562	electron-transport		ECL_02291
preprotein translocase subunit SecD	transport	secD	ECL_01165
**MNV Downregulated by at least 1 log_2 fold change**			
**Identified Proteins**	**Function**	**Gene Name**	**Gene Locus ID**
succinate semialdehyde dehydrogenase	catabolism		ECL_04622
fructose-1,6-bisphosphatase	gluconeogenesis	fbp	ECL_00637
putative fimbrial protein	cell adhesion	*fimF*	ECL_01283
OmpA family protein	transport		ECL_03936

Table S3. Proteins uniquely absent or present in MNV OMVs.

Absent in MNV OMVs		Present in MNV OMVs	
**Identified Proteins**	**Function**	**Identified Proteins**	**Function**
2-amino-3-ketobutyrate CoA ligase	biosynthetic process	chlorinating enzyme	biosynthetic process
acetate kinase	biosynthetic process	ADP-L-glycero-D-manno-heptose-6-epimerase	biosynthetic process
2,5-diketo-D-gluconate reductase A	L-ascorbic acid biosynthetic process	cysteine synthase A	cysteine biosynthetic process
3-octaprenyl-4-hydroxybenzoate carboxy-lyase	ubiquinone biosynthetic process	agmatinase	biosynthetic process
ketol-acid reductoisomerase	isoleucine and valine biosynthetic process	acetyl-CoA carboxylase, carboxyl transferase subunit beta	fatty acid biosynthesis
dihydropteroate synthase	folic acid biosynthetic process	carbamoyl-phosphate synthase small subunit	biosynthetic process
UTP-glucose-1-phosphate uridylyltransferase	lipopolysaccharide biosynthetic process	ATP-dependent protease	biosynthetic process
ribose transport system substrate-binding protein	transport	spermidine/putrescine transport system substrate-binding protein	transport
putative outer membrane usher protein	transport	ribose transport system substrate-binding protein	transport
putative glycine/betaine/choline ABC transporter substrate-binding component	transport	glycine betaine/proline transport system substrate-binding protein	transport
extracellular solute-binding protein	transport	extracellular ligand-binding receptor	transport
copper resistance protein B (plasmid)	cellular copper ion homeostasis	arginine transporter, periplasmic binding protein	transport

Ferric aerobactin receptor	iron ion homeostasis	hypothetical protein ECL_00179	transport
hypothetical protein ECL_02069	stress response	bacterioferritin	transport, homeostasis
universal stress protein	stress response	copper resistant protein PcoB	homeostasis
silver binding protein precursor SilE (plasmid)	stress response	Ferric aerobactin receptor	iron ion homeostasis
OsmC family protein	stress response	copper resistance protein B (plasmid)	cellular copper ion homeostasis
single-strand DNA-binding protein Ssb	DNA replication and repair	RNA polymerase factor sigma-32	stress response
uracil phosphoribosyltransferase	nucleoside metabolic process	putative catalase	stress response
flagellar basal body rod protein FlgG	cell motility	methylmalonate-semialdehyde dehydrogenase	oxidoreductase
fructose-1,6-bisphosphatase	gluconeogenesis	glycine dehydrogenase	oxidoreductase
PTS system glucose-specific EIICB component	PEP dependent sugar phosphotransferase system	alcohol dehydrogenase	oxidoreductase
carbamate kinase	metabolism	cytochrome o ubiquinol oxidase subunit II	oxidoreductase
nucleoside triphosphatase, D5 family, partial	hydrolase	NADH:ubiquinone oxidoreductase, chain C,D	oxidoreductase
ATP-dependent Clp protease proteolytic subunit	hydrolase	cytochrome d ubiquinol oxidase subunit III	oxidoreductase
hypothetical protein ECL_02463	oxidoreductase	5-carboxymethyl-2-hydroxymuconate semialdehyde dehydrogenase	oxidoreductase
transcription termination factor Rho	transcription	cytochrome bd-I oxidase subunit II	oxidoreductase

translation initiation factor IF-3	translation	murein hydrolase B	hydrolase
tyrosyl-tRNA synthetase	aminoacylation	serine endoprotease	hydrolase
rod shape-determining protein mreB	determines cell shape	ATP-dependent OLD family endonuclease	hydrolase
Flagellar basal-body rod protein, FlgC	cell motility	bifunctional UDP-sugar hydrolase/5'-nucleotidase periplasmic	nucleotide catabolism, hydrolase
putative fimbrial protein	Cell adhesion	3-ketoacyl-CoA thiolase	Fatty acid beta oxidation
hypothetical protein ECL_04497	unknown	multifunctional fatty acid oxidation complex subunit alpha	Fatty acid beta oxidation
putative outer membrane lipoprotein	unknown	acyl-CoA dehydrogenase	Fatty acid beta oxidation
hypothetical protein ECL_02480	unknown	50S ribosomal protein L16	translation
hypothetical protein ECL_00450	unknown	translation initiation factor IF-2	translation
capsid scaffold-like protein	phage protein	30S ribosomal protein S10	translation
		50S ribosomal protein L14	translation
		50S ribosomal protein L6	translation
		maltodextrin phosphorylase	carbohydrate metabolism
		2,3-bisphosphoglycerate-independent phosphoglycerate mutase	glycolysis, gluconeogenesis
		malate dehydrogenase	TCA, metabolism
		Aspartate ammonia-lyase	TCA
		PTS system mannitol-specific transporter subunit IIC	PEP dependent sugar phosphotransferase system

peptidyl-prolyl cis-trans isomerase	protein folding
putative sensory transduction regulator	unstable protein stabilizer
ATPase	chaperone
beta-ketoacyl-ACP synthase	lipid metabolism
antimicrobial peptide ABC transporter	lipid metabolism
replicative DNA helicase	DNA replication
succinylglutamic semialdehyde dehydrogenase	catabolism
L-threonine 3-dehydrogenase	catabolism
murein transglycosylase C	PG metabolism
hypothetical protein ECL_01167	enzymatic
non-heme chloroperoxidase	enzymatic
putative ureidoglycine-glyoxylate aminotransferase	enzymatic
putative S-transferase	enzymatic
putative short-chain alcohol dehydrogenase	enzymatic
two-component response regulator	transcription regulation
ecotin	protease inhibitor
lipoyl synthase	protein lipoylation
DNA polymerase III subunit alpha	DNA replication
hypothetical protein ECL_04364	biofilm formation

	conjugal transfer mating pair stabilization protein TraG (plasmid)	cell interactions
	hypothetical protein ECL_03858	cell shape regualtion
	flagellar hook-associated 2 domain-containing protein	cell motility, cell adhesion

Table S4. Bacterial isolates used in experiments.

Name	ATCC Number	Source
Enterobacter cloacae (EC)	13047	ATCC
Bacteroides thetaiotaomicron (BT)	29148	Dr. Stephanie Karst, University of Florida
Lactobacillus acidophilus (LA)	4356	Dr. Graciela Lorca, University of Florida

Table S5. Primers used for RT-qPCR validation.

Gene	Sequence (5'->3')
rpoB-Fwd	TACGCGTCGACCCAACCAAC
rpoB-Rev	TCGATCTCATCGCGCAGCAG
groL-Fwd	CCAACAACGTGAAAGCGGGC
groL-Rev	GCCGCATACTGCAGAGCAGA
cgsD-Fwd	ACCGCGAGAAAGAGATCCTG
cgsD-Rev	ACCGCCTGAGTTCGGTTTTT
fadB-Fwd	CGCAGGTAAAGATGTTGGCG
fadB-Rev	GTTTGGCCTTCCAGTCGAGA
flgB-Fwd	GCGCCCACGACCGATTTACT
flgB-Rev	ACGCTCCCGGTCCATATCCA
fliI-Fwd	GGCCGAAGCCGGACACTATC
fliI-Rev	GTTGCGCTGGAAGCTGGAGA
ftsL-Fwd	TAGGAAGCAACGAGCGCCAT
ftsL-Rev	GCAGAGTGGCAGCTTCCCAA
ompA family-Fwd	GCAGCGGTGACAACATCATC
OmpA family-Rev	ATCACATTCACGGCGGTCTT
ompX-Fwd	ACGACTGGGCAAGCATCTAC
ompX-Rev	GAGAAGCCGTAGTCGCTGTT
rcsC-Fwd	GGCTGGCAATTTGTGAGAAG
rcsC-Rev	GCGCAGAATAGAGCGGAATA
